# Secreted Factors and EV-miRNAs Orchestrate the Healing Capacity of Adipose Mesenchymal Stem Cells for the Treatment of Knee Osteoarthritis

**DOI:** 10.3390/ijms21051582

**Published:** 2020-02-26

**Authors:** Enrico Ragni, Carlotta Perucca Orfei, Paola De Luca, Alessandra Colombini, Marco Viganò, Laura de Girolamo

**Affiliations:** Laboratorio di Biotecnologie Applicate all’Ortopedia, IRCCS Istituto Ortopedico Galeazzi, Via R. Galeazzi 4, I-20161 Milano, Italy; enrico.ragni@grupposandonato.it (E.R.); deluca.paola@grupposandonato.it (P.D.L.); alessandra.colombini@grupposandonato.it (A.C.); marco.vigano@grupposandonato.it (M.V.); laura.degirolamo@grupposandonato.it (L.d.G.)

**Keywords:** MSCs, fat tissue, osteoarthritis, cytokines, extracellular vesicles, miRNAs, joint

## Abstract

Mesenchymal stem cells (MSCs) derived from adipose tissue and used either as expanded cells or minimally manipulated cell preparations showed positive clinical outcomes in regenerative medicine approaches based on tissue restoration and inflammation control, like in osteoarthritis (OA). Recently, MSCs’ healing capacity has been ascribed to the large array of soluble factors, including soluble cytokines/chemokines and miRNAs conveyed within extracellular vesicles (EVs). Therefore, in this study, 200 secreted cytokines, chemokines and growth factors via ELISA, together with EV-embedded miRNAs via high-throughput techniques, were scored in adipose-derived MSCs (ASCs) cultivated under inflammatory conditions, mimicking OA synovial fluid. Both factors (through most abundantly expressed TIMP1, TIMP2, PLG and CTSS) and miRNAs (miR-24-3p, miR-222-3p and miR-193b-3p) suggested a strong capacity for ASCs to reduce matrix degradation activities, as those activated in OA cartilage, and switch synovial macrophages, often characterized by an M1 inflammatory polarization, towards an M2 phenotype. Moreover, the crucial importance of selecting the target tissue is discussed, showing how a focused search may greatly improve potency prediction and explain clinical outcomes. In conclusion, herein presented data shed light about the way ASCs regulate cell homeostasis and regenerative pathways in an OA-resembling environment, therefore suggesting a rationale for the use of MSC-enriched clinical products, such as stromal vascular fraction and microfragmented adipose tissue, in joint pathologies.

## 1. Introduction

Osteoarthritis (OA) is a heterogeneous disease with a wide range of underlying pathways and involves structural alterations mainly in the articular cartilage, subchondral bone and synovial membrane [1]. In particular, OA is characterized by a complex biological imbalance between the repair and destruction of joint tissues, rather than a stand-alone mechanical degeneration [2]. The initial loss of cartilage integrity leads first to superficial erosions and later to fissures followed by the expansion of calcifications. Matrix degradation products and proinflammatory mediators deregulate chondrocyte function and stimulate synovium proliferative and inflammatory responses of both resident synoviocytes and macrophages. Eventually, bone turnover is increased with the development of subchondral bone marrow lesions [3].

Due to the strong biological fingerprint, the ideal OA management should rely on the restoration of tissue homeostasis. Nevertheless, to date, available treatments are not supposed to have a direct influence on the damaged tissues but, rather, on symptoms control. Oral nonsteroidal anti-inflammatory and analgesic drugs, as well as intra-articular corticosteroids or hyaluronic acid (HA), are commonly used alone or in combination to manage early pain and improve mobility [4]. In end-stage OA, joint replacement is still the most effective practice [5,6]. Nevertheless, although total knee replacement showed greater pain relief and functional improvement than nonsurgical treatments [7], up to 25% of patients still complain of pain and disability after surgery [8]. In this perspective, new biological-based products aimed to prevent the progression of OA and restoring tissue homeostasis have been introduced. They are mostly based on mesenchymal stem cells (MSCs) whose pro-regenerative properties are well known. Of note, in addition to expanded MSCs, MSC-enriched products prepared at the point of care under minimal manipulation are also available, both from bone marrow (bone marrow aspirate concentrate—BMAC) and adipose tissue (stromal vascular fraction—SVF or microfragmented fat tissue—MFAT).

MSCs are multipotent cells with a high capacity for self-renewal that, in adult life, are found dormant in vivo as pericytes and, once activated, take part in tissue repair [9]. Further, signals associated with disease, such as pro-inflammatory mediators in the joint cavity, may enhance MSCs activity, which in turn secrete factors, chemokines and cytokines able to limit the extent of damage and improve tissue healing [10]. In this frame, both in vitro and in vivo, MSCs were shown to secrete an array of molecules with, among others, anti-inflammatory properties, such as: prostaglandin-E2 (PGE2), responsible for inducing an M2 switch in human synovial macrophages [11]; indoleamine 2,3-dioxygenase (IDO), suppressing T-cell proliferation [12] and driving M2 polarization of macrophages [13] and other anti-inflammatory and immunosuppressive molecules, like iNOS, TSG6, HO1, TGF-β and galectins [14]. Supporting this notion, both MSCs and MSC-conditioned mediums (CMs) have been shown to have a protective effect on cartilage in a murine OA model, together with pain reduction [15], the last due to a decrement of inflammation, as also shown in both in vitro models of OA chondrocyte [16] and ex vivo explants of cartilage and synovia [17]. In fact, together with cartilage destruction, inflammation is a crucial player in the establishment and maintenance of OA and positively correlates with knee pain [18], with synovial membrane inflammation a hallmark of the pathology [19]. Moreover, higher levels of pro-inflammatory cytokines and infiltrated immune cells, predominantly macrophages, positively correlate with OA severity [20].

Together with secreted factors, in the last years, it has been proposed that also extracellular vesicles (EVs) and embedded molecules, such as proteins, lipids or nucleic acids (predominantly miRNAs), may contribute to MSC pro-regenerative and immunosuppressive potential [21,22]. In OA, preliminary in vitro results showed MSC-EVs protective effects on both chondrocytes and synoviocytes [23,24]. Notably, EVs derived from synovial MSCs overexpressing miR-140-5p induced proliferation of chondrocytes in vitro, supporting the crucial role of miRNAs [25]. Consistently, in an OA mouse model, synovium MSC-EVs attenuated disease scores by promoting chondrocyte proliferation and migration [26]. Similar results were obtained with EVs obtained from ASCs, bone marrow- and amniotic fluid-derived MSCs, with an almost complete restoration of cartilage, less pain and a M1 to M2 polarization of macrophages [27,28,29]. In this frame, MSC-EVs have been demonstrated to possess regulatory functions on immune cells by regulating the proliferation, maturation, polarization and migration of various immune effector cells [30]. As an example, in a model of collagen-induced OA, MSC-EVs exerted anti-inflammatory effects through the inhibition of B cell maturation [27]. Moreover, an increasing number of reports showed the M1-suppressing and M2-enhancing capacities of MSC-EVs on macrophages, mainly through shuttled miRNAs [30], contributing to the beneficial effect of ASCs on macrophage regulation [31]. Intriguingly, also MFAT was shown to switch off synovial macrophage activity in vitro [32], through a decrease of activating chemokines and, possibly, the presence of M2-modulating EVs.

Therefore, a complex network of both soluble factors and EVs-embedded miRNAs would be responsible for the promising results of MSC-based therapies in the OA setting. Nevertheless, to date, a complete picture of secreted molecules and miRNAs is missing, especially in conditions mimicking those MSCs find once injected in the OA joint. In fact, often, the experimentally induced inflammatory conditions are very far from the real amount and types of inflammatory factors found in a pathological joint. The aim of this study was therefore to identify secreted cytokines, chemokines and growth factors and EV-shuttled miRNAs from ASCs treated with inflammatory mediators at concentrations resembling those in the OA synovial fluid based on literature reports [33,34,35,36,37,38,39,40,41] in order to clarify how the ASCs regulate cell homeostasis and regenerative pathways in OA joints.

## 2. Results

### 2.1. ASCs Characterization

**Figure 1:**
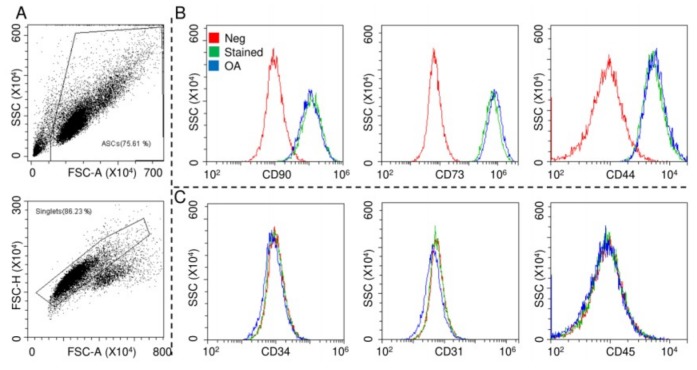
Surface marker expressions of adipose-derived MSCs (ASCs). After FSC/SSC gating and doublets removal (**A**), ASCs without and with osteoarthritis (OA) inflammation resulted positive for CD90/73/44 MSC markers (**B**) and negative for CD34/31/45 hemato/endothelial determinants (**C**). Red: unstained ASCs, green: stained ASCs and blue: stained inflamed (OA) ASCs. Representative cytograms of a single population are shown.

ASCs were characterized by flow cytometry for both MSCs and hemato-endothelial markers in the absence and presence of OA-resembling cytokines TNFα (5 pg/mL) + IL1β (10 pg/mL) + IFNγ (40 pg/mL), with working concentrations obtained by calculating the median values from the OA synovial fluid analysis [33,34,35,36,37,38,39,40,41] (Figure 1A). The cells were strongly positive for mesenchymal epitopes (Figure 1B, CD90-CD73-CD44) and negative for CD31, CD34 and CD45, confirming their identity (Figure 1C). Inflammation did not alter marker expression, as well as viability (95% ± 1% in control ASCs and 96% ± 1% in the OA condition).

### 2.2. ASCs Secreted Factors

**Figure 2:**
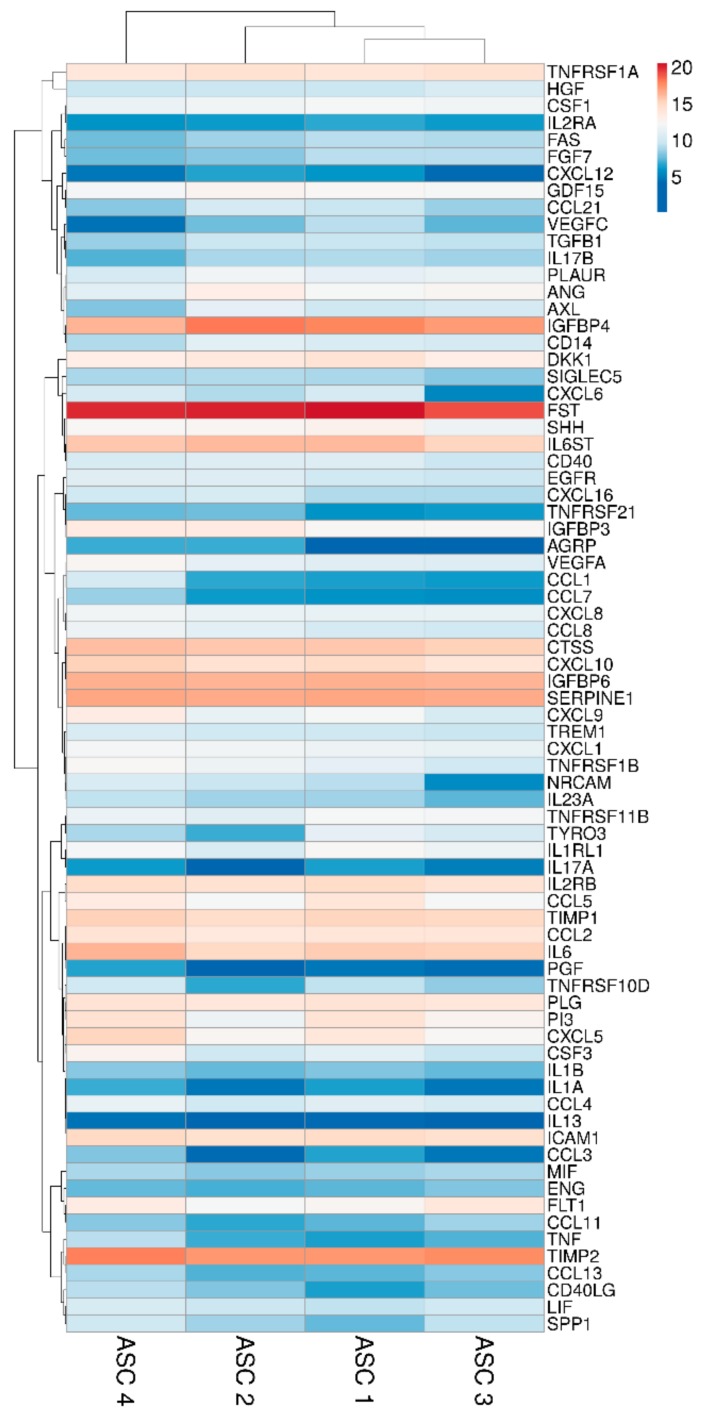
Heat map of ASCs-secreted factors across all samples. Heat map of hierarchical clustering analysis: the log_2_ values of 75 detected secreted factors after normalization per 10^6^ ASCs. The sample clustering tree is shown at the top. The color scale shown in the map illustrates the absolute expression levels of factors across all samples: red shades = high expression levels (high log_2_(pg) per 10^6^ ASCs) and blue shades = lower expression levels (low log_2_(pg) per 10^6^ ASCs).

Two hundred molecules, including inflammatory mediators and growth factors, chemokines, receptors and cytokines, were tested in conditioned medium produced by ASCs under OA-simulating conditions. Seventy-five molecules were scored in all samples at 48 h of induction (Table 1). Interestingly, about 0.2 ng of IL1β and TNFα were detected. This value is higher than the initial levels in the culture medium provided by the medium supplementation (0.01 and 0.05, respectively). On the other side, IFNγ was below detection, meaning a consumption of the administered cytokine without further release given by inflammation at the selected time point of 48 h, being aware that a more prolonged inflammation might lead to its secretion. Hierarchical clustering was able to show a higher similarity for ASC 1 and 3 (Figure 2), although in a pattern of overall conservation, since the correlation analysis showed a mean R^2^ of 0.96 ± 0.02. Consequently, an average intensity value was calculated in order to provide a guide to the level of each factor (Table 1). In 48 h, per million cells, four factors were secreted with an average amount superior to 100 ng, namely FST (mean 1037 ± SD 377), TIMP2 (196 ± 43), IGFBP4 (182 ± 85) and SERPINE1 (108 ± 9). Fourteen factors were between 10 and 100 ng: IGFBP6 (86 ± 7), IL6ST (56 ± 15), CTSS (52 ± 10), IL6 (49 ± 23), TIMP1 (34 ± 6), ICAM 1 (27 ± 5), CXCL10 (26 ± 10), IL2RB (24 ± 5), TNFRSF1A (18 ± 4), CCL2 (16 ± 2), PLG (16 ± 2), CXCL5 (16 ± 15), DKK1 (12 ± 4) and PI3 (12 ± 9). Eventually, the average amount of 24 factors was between 10 and 1 ng and, of the other 33, below 1 ng.

**Figure 3:**
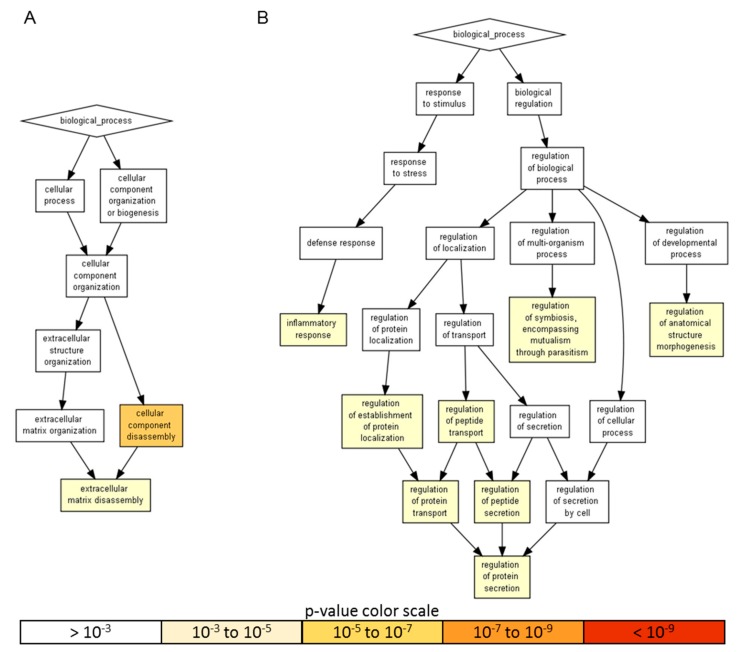
A visualization of the biological process gene ontology annotations using Gorilla for identified secreted factors. **A**) The background dataset used is composed of 200 human factors tested in the ELISA arrays (see Materials and Methods). Enrichment using the 18 factors expressed at > 10 ng per 10^6^ ASCs is shown. **B**) Enrichment using the 57 factors expressed at < 10 ng per 10^6^ ASCs is shown. The Gorilla settings were left at default values: *p*-value threshold of *p* < 10^−3^, organism Homo sapiens.

Gene ontology enrichment analysis was performed using annotations for the 200 factors as the background dataset. Two groups were scored: (i) the 18 factors with an amount > than 10 ng, representing a more focused and preponderant secretome fingerprint and (ii) the factors secreted at lower levels that, even if less abundant, could represent an equally important overall signal due to higher number of molecules. For heavily secreted factors, gene ontology (GO) enrichment analysis identified two significant (*p*-value < 10^−3^) biological process (BP) terms, namely cellular component disassembly (GO:0022411), encompassed by five proteins, and extracellular matrix disassembly (GO:0022617), defined by four players (Figure 3A and Table 2). When the other 57 factors were analyzed, eight statistically enriched BPs were found (Figure 3B and Table 2), with the terminal nodes being for the: (i) biological regulation pathway—regulation of anatomical structure morphogenesis (GO:0022603, 28 molecules), regulation of symbiosis, encompassing mutualism through parasitism (GO:0043903, seven) and regulation of protein secretion (GO:0050708, 19) and, for the (ii) response to stimulus cascade—inflammatory response (GO:0006954, 31). Further, due to the reported role of ASCs in modulating synovial macrophages, the inflammatory response list was sifted through a Panther algorithm to identify specific and macrophage-related GO terms. A few terms emerged: macrophage chemotaxis (GO:0048246, two factors) and migration (GO:1905517), both defined by CCL3 and CCL5; the positive regulation of macrophage differentiation (GO:0045651) and regulation of macrophage differentiation (GO:0045649), both defined by CSF1 and TGFB1; the positive regulation of macrophage chemotaxis (GO:0010759, CCL5 and CSF1) and regulation of macrophage chemotaxis (GO:0010758, CSF1, CCL5 and MIF) and the positive regulation of macrophage activation (GO:0043032, CCL3 and IL13) and regulation of macrophage activation (GO:0043030, CCL3, IL13 and MIF). Eventually, to have a comprehensive overview of the entire dataset, in the inflammatory response terms, nine proteins of the > 10 ng group also fell (CCL2, CXCL10, CXCL5, EGFR, ICAM1, IGFBP4, IL6, TIMP1 and TNFRSF1A). CCL2 belonged to macrophage chemotaxis, LIF to the regulation of macrophage differentiation and IL1RL1 and IL6 to macrophage activation. Related to macrophages and their ability to migrate, monocyte chemotaxis (GO:0002548, IL6 and CCL2) emerged. Interestingly, other GO terms related with motility were identified, like leukocyte chemotaxis (GO:0030595, IL6, CCL2, CXCL5 and CXCL10); lymphocyte chemotaxis (GO:0048247, CCL2 and CXCL10) and granulocyte chemotaxis (GO:0071621, CCL2, CXCL5 and CXCL10). In particular, the positive regulation of leukocyte migration (GO:0002687, ICAM1, IL6 and CXCL10) term was defined, indicating the influence of highly secreted factors on processes that activate or increase the frequency, rate or extent of leukocyte migration. Supporting this notion, a more general analysis of the 65 factors allowed to identify 23 molecules under leukocyte chemotaxis (GO:0030595), with only two factors related to the negative regulation of leukocyte chemotaxis (GO:0002689, MIF and CCL2) and 16 to the positive regulation of leukocyte chemotaxis (GO:0002690). Therefore, overall, ASC secretome was enriched in molecules involved in both extracellular matrix homeostasis and the inflammatory response control to different levels, from migration to homeostasis/activation.

### 2.3. Characterization of ASC-Derived Extracellular Vesicles

**Figure 4:**
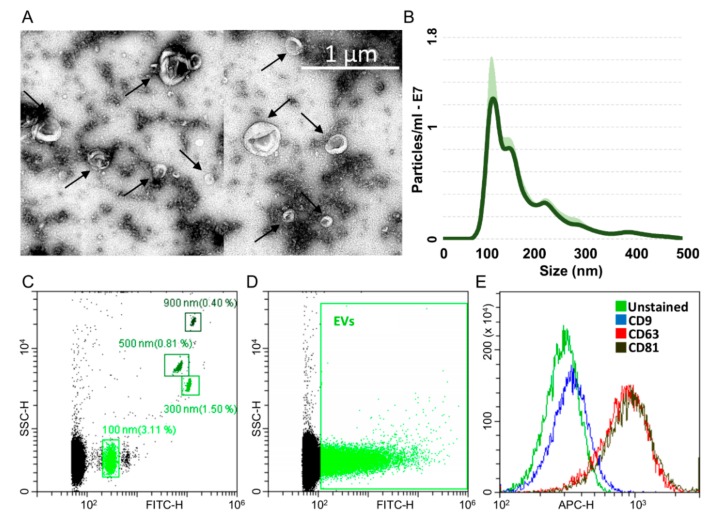
Characterization of ASC-EVs (extracellular vesicles). **A**) Transmission electron micrographs of ASC-EVs showing particles with characteristic cup-shaped morphology. **B**) Size distribution of nanoparticles by NanoSight particle-tracking analysis. **C**) Nanometric fluorescent beads with diameters of 100 nm, 300 nm, 500 nm and 900 nm used for flow cytometer calibration confirmed instrument sensitivity with respect to background. **D**) CFSE-stained ASC-EVs (green) with respect to background. **E**) Flow cytometry scoring CD63/81 EV marker positivity on CFSE-labeled ASC-EVs. CD9 resulted barely positive. One representative donor is shown.

ASCs treated with OA cytokines for 48 h release around 22,500 extracellular vesicles (EVs) per cell. Isolated EVs exhibited the characteristic cup-shape morphology (Figure 4A) and were within the literature-reported size range (50–400 nm in diameter), with enrichment in the small particles (mode 118 ± 19 nm) (Figure 4B). To detect particles within the nanometer range, the flow cytometer has been calibrated with FITC-fluorescent beads of predetermined size (100 to 900 nm, Figure 4C). Purified EVs felt within the 100 nm size, confirming NTA data (Figure 4D). EVs were strongly positive for extracellular vesicle CD63 and CD81 markers (Figure 4E), 78.9% ± 1% and 81.6% ± 0.9%, respectively, consistent with previously reported characteristics of extracellular vesicles. On the contrary, CD9 staining was low (2.4% ± 0.2%).

### 2.4. EV-Associated miRNAs

**Figure 5:**
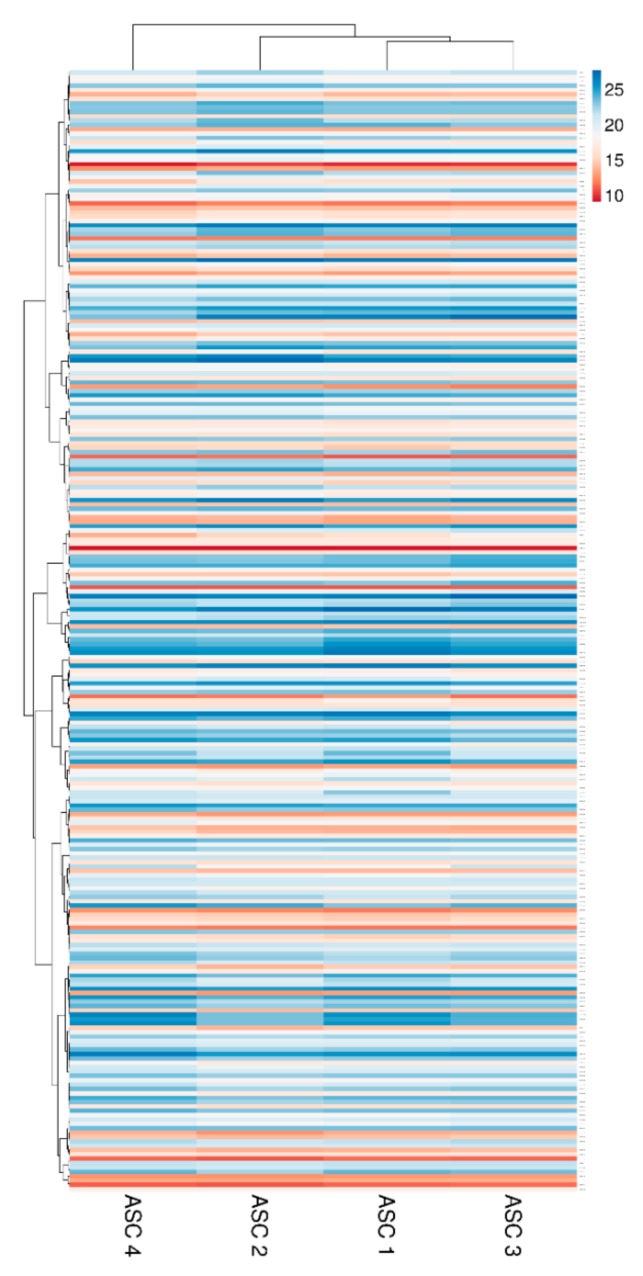
Heat map of EV-miRNAs across all samples. Heat map of hierarchical clustering analysis of the normalized C_RT_ values of detected miRNAs in ASC-EVs. Rows are centered. The sample clustering tree is shown at the top. The color scale shown in the map illustrates the absolute expression levels of factors across all samples: red shades = high expression levels (low C_RT_ values) and blue shades = lower expression levels (high C_RT_ values).

Two-hundred and fifty-seven miRNAs were identified as EVs embedded at various levels of expression in all the four ASCs under analysis (Supplementary Table S1). EV-miRNA hierarchical clustering output was superimposable to the one obtained for factors with higher similarity for ASC 1 and 3 (Figure 5). Nevertheless, a pattern of overall conservation was again present, with correlation analysis showing a mean R^2^ of 0.97 ± 0.01. Therefore, an average C_RT_ value was calculated in order to provide a guide to the level of each miRNA (Supplementary Table S1).

**Figure 6:**
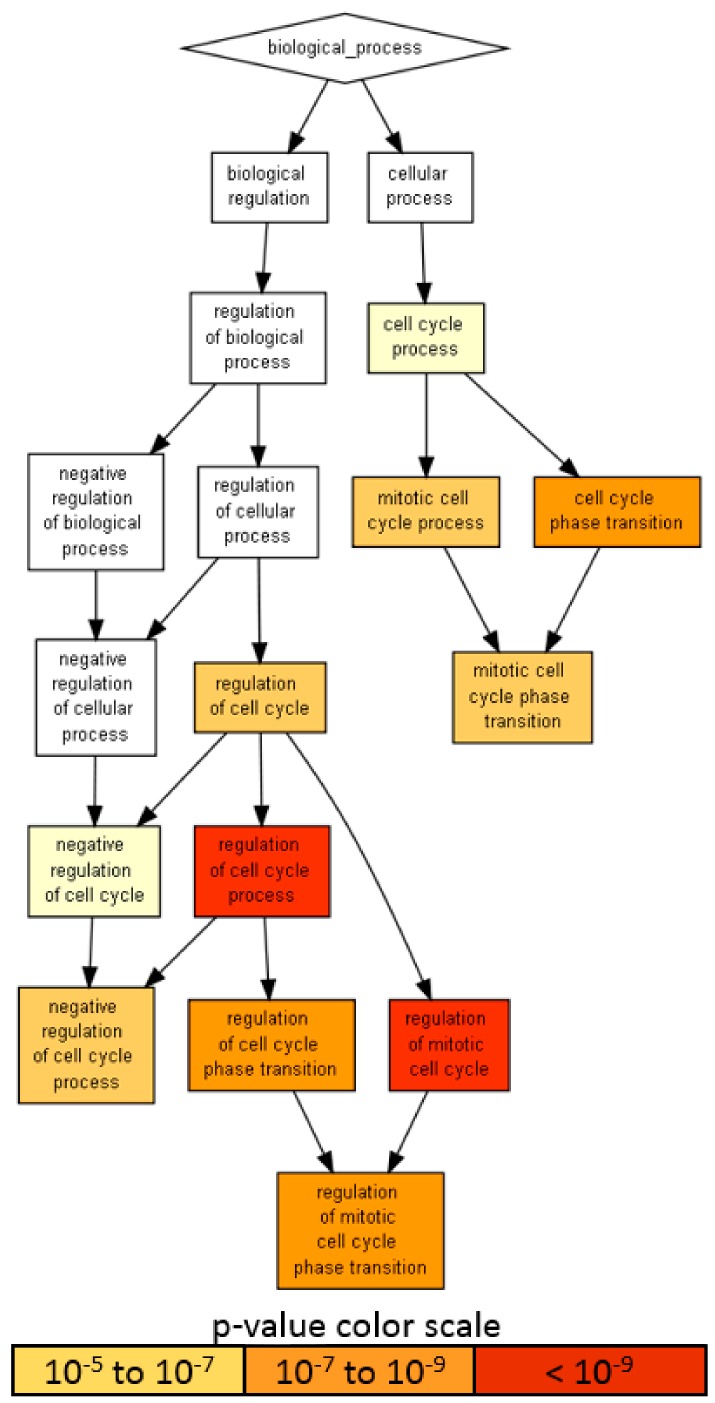
A visualization of the biological process gene ontology annotations using Gorilla for targets of abundant EV-miRNAs. The background dataset used is composed of the entire human genome. Enrichment using the 818 mRNAs verified as targets of EV-miRNAs in the first quartile of expression. The Gorilla settings were left at default values: *p*-value threshold of *p* < 10^−6^, organism Homo sapiens.

Given the presence on average of one abundant miRNA molecule per EV in MSC, 100 MSC-EVs would be needed to shuttle its genetic message to target cells [42,43]. Therefore, only highly abundant miRNAs laying in the first quartile of expression were considered for further analysis (65 miRNAs, covering 96.7% of the genetic message) (Supplementary Table S1, yellow background). Ingenuity pathway analysis (IPA) of the 65 miRNAs used to score only experimentally observed targets identified 818 mRNAs (Supplementary Table S2). An unbiased GO enrichment analysis against the whole genome, with a stringent *p*-value threshold (< 10^−6^) due to the high number of scored mRNAs, was able to identify BP related to cell cycle regulation (Figure 6), with the most significantly (*p*-value < 10^−9^) enriched terms being: regulation of the cell cycle process (GO:0010564, *p*-value 1.31 × 10^−11^, 45 associated genes) and regulation of the mitotic cell cycle (GO:0007346, 8.14 × 10^−10^, 39 genes). Notably, negative regulation of the cell cycle process term (GO:0010948, 6.12 × 10^−7^, 24 genes) suggested that shuttled miRNAs may reduce the activity of genes delaying the cell cycle events, thus promoting proliferation. To define better this influence, the enriched molecular function (MF) GO search was able to specify target terms as the cyclin-dependent protein serine/threonine kinase regulator activity term (GO:0016538, 2.02 × 10^−9^), encompassing several cyclins (*CCNA2*, *CCNE2*, *CCNF*, *CCNE1*, *CCND1* and *CCND3*); cyclin-dependent kinases (*CDK4*) and inhibitors (*CDKN2A*, *CDKN1C*, *CDKN1B* and *CDKN1A*) and an apoptosis-related cysteine peptidase (*CASP3*).

### 2.5. Target and Effect Prediction of EV-miRNAs on OA-Cartilage

To frame the power of EV-miRNAs in the OA setting, the 818 target mRNAs were filtered through 2368 abundantly expressed genes, identified as laying in the first quartile of expression (out of 9474 total genes), in cartilage biopsies from OA patients [44]. After filtering, 224 EV-miRNA targets were left and shared (Supplementary Table S3). First, identified genes were searched against those in the IPA database of the OA-related pathway. Nineteen genes resulted targets of EV-miRNAs (*ATF4*, *BMPR2*, *FGF2*, *FGFR1*, *FGFR3*, *FN1*, *HIF1A*, *ITGA5*, *JAG1*, *MEF2C*, *MMP3*, *SMAD3*, *SMAD4*, *SMAD5*, *SP1*, *TGFBR1*, *TGFBR2*, *TIMP3* and VEGFA). Most represented protein classes were transcription factors (PC00218: HIF1A, MEF2C, *SMAD3*/4/5 and *SP1*); receptors (PC00197: BMPR2 and TGFBR1/2) and signaling molecules (PC00207: *FGF2*, *FN1* and *VEGFA*). Notably, many of these molecules specified molecular cascades that are directly involved with OA insurgence and development, like TGF-beta (P00052: *BMPR2*, *SMAD3/4/5* and *TGFBR1/2*); FGF (P00021: *FGF2* and *FGFR1/3*) and Wnt (P00057: *SMAD4/5* and *TGFBR1*) signaling pathways. Second, GO enrichment (*p*-value < 10^−6^ for enriched GO term threshold) was conducted by scoring the 224 mRNAs against the entire first quartile of OA cartilage as background. Several BP terms were identified, suggesting that when a tissue and/or disease-focused search is performed, the specificity and power of the outcomes increase (Supplementary Figure S1). In particular, five terms were more significantly enriched (*p*-value < 10^−9^): the negative regulation of multicellular organismal process (GO:0051241, 1.68 × 10^−10^, 52 genes); regulation of cellular component movement (GO:0051270, 3.93 × 10^−10^, 50 genes); regulation of cell motility (GO:2000145, 4.42 × 10^−10^, 48 genes); cellular response to chemical stimulus (GO:0070887, 7.71 × 10^−10^, 68 genes) and regulation of cell migration (GO:0030334, 8.45 × 10^−10^, 46 genes). Interestingly, EV-miRNAs seem to selectively target genes defining the positive regulation of movement: positive regulation of cell migration (GO:0030335, 1.37 × 10^−9^, 35 genes); positive regulation of cell motility (GO:2000147, 2.76 × 10^−9^, 35 genes) and positive regulation of locomotion (GO:0040017, 6.76 × 10^−9^, 35 genes). All these BP regulating the cell response and adaptation relies on several cascades ending in a complex transcriptional regulation, as outlined by the enriched MF GO term DNA-binding transcription factor activity, RNA polymerase II-specific (GO:0000981, 1.79 × 10^−8^, 38 genes).

**Figure 7:**
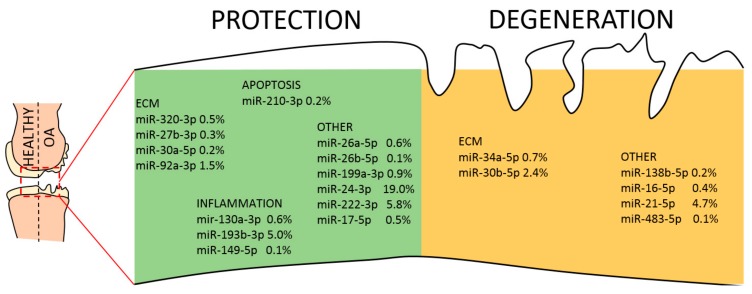
EV-miRNAs involved in protective or destructive mechanisms in the OA joint. Indication of miRNAs in the first quartile of expression in ASC-EVs and their role in cartilage homeostasis. miRNAs are divided per category and the relative amount of their genetic message shown.

Finally, the list of 65 abundant EV-miRNAs was compared with the database of miRNAs reported to be directly involved in OA pathogenesis, both protective and destructive [45]. Seventeen miRNAs resulted to be involved with protective mechanisms, seven regulate degenerative pathways and four are controlling both mechanisms (Table 3 and Figure 7). Interestingly, scoring the genetic weight of the EV-shuttle and miRNA-related message, protective molecules account for almost 40%, whereas degenerative ones less than 10%. Overall, this might indicate a preponderance of EV-miRNAs in cartilage and synovium protection.

### 2.6. Target and Effect Prediction of EV-miRNAs on Synovial Macrophages

In a recent publication, the transcriptional landscape of infiltrated synovial macrophages has been defined in OA patients [46]. Two types of macrophages were identified: inflammatory-like OA (iOA) macrophages, which are closely aligned to rheumatoid arthritis (RA) macrophages, and classical OA (cOA) ones displaying, together with lower inflammatory features, aberrant tissue repair mechanisms and secretion of factors that may directly contribute to cartilage degradation. Since, in a previous publication, the macrophage transcriptional fingerprint was able to clearly distinguish RA and OA cells [47], we opted to consider newly defined cOA macrophages’ mRNA dataset for further analysis. Eight-hundred and eighteen EV-miRNA target mRNAs were filtered against the first quartile of expression (2498 transcripts) of synovial macrophages (total of 9991), and 214 shared genes were identified (Supplementary Table S4). Only 112 genes were shared between the OA cartilage and synovial macrophages, suggesting a divergent response.

First, identified genes were searched against those in the IPA database of the inhibition of metalloproteases pathway, a subclass of the previously scored OA pathway, given the role of synovial macrophages in cartilage ECM destruction. Interestingly, *MMP1*, *3* and *9* resulted experimentally verified targets, together with *RECK* and *TIMP3* inhibitors, suggesting a profound role of EVs on OA macrophages remodeling activity. Further, searching the GO term macrophage activation (GO:0042116), out of 94 annotated genes, six emerged as possible targets (*APP*, *IL10*, *JUN*, *TLR4*/7 and *TNFa*). Intriguingly, abundant miRNAs did not target directly the expression of canonical M1 chemokines (CCL2/3/4/8/20 in the top expressed OA-synovial macrophages mRNAs) and cytokines (IL1A/B), with the exception of IL6 and TNFa. On the contrary, EVs might regulate macrophage polarization by targeting TLR4 that, through NF-kb, suppresses STAT3, whose predominance over STAT1 increases M2 polarization, associated with immune tolerance and tissue repairing. Then, to have a more comprehensive view, the genes were scored against the OA synovial-macrophages first quartile mRNAs (*p*-value < 10^−6^) as background to identify enriched GO terms. Again, several BP emerged, with a completely different pattern with respect to cartilage. In particular, six terms were strongly enriched (*p*-value < 10^−9^): the anatomical structure development (GO:0048856, 6.05 × 10^−12^, 84 genes); animal organ development (GO:0048513, 3.66 × 10^−11^, 46 genes); positive regulation of multicellular organismal process (GO:0051240, 1.51 × 10^−10^, 68 genes); negative regulation of cell death (GO:0060548, 4.49 × 10^−10^, 54 genes); regulation of smooth muscle cell proliferation (GO:0048660, 7.47 × 10^−10^, 19 genes) and negative regulation of programmed cell death (GO:0043069, 9.95 × 10^−10^, 50 genes). Interestingly, and differently from cartilage, in macrophages, EV-miRNAs may preferentially target genes regulating apoptosis and cell death-related pathways, as emphasized by other significantly enriched GO terms like the negative regulation of the apoptotic process (GO:0043066, 1.47 × 10^−9^, 1.81 × 10^−6^, 49 genes) and regulation of cell death (GO:0010941, 6.29 × 10^−9^, 71 genes). In fact, narrowing the GO enrichment analysis (*p*-value < 10^−9^ as the threshold for GO enrichment), cell death and its regulation emerged as a main constituent of a GO term branch (Supplementary Figure S2). Further, EV-miRNAs are also able to target genes defining myeloid lineage, as shown by GO terms: the regulation of myeloid cell differentiation (GO:0045637, 1.87 × 10^−7^, 19 genes) and positive regulation of myeloid cell differentiation (GO:0045639, 2.04 × 10^−7^, 13 genes). As for cartilage, the different BPs rely on a high number of molecular and transcriptional cascades, as emphasized by the MF GO term DNA-binding transcription factor activity, RNA polymerase II-specific (GO:0000981, 8.45 × 10^−9^, 31 genes).

**Figure 8:**
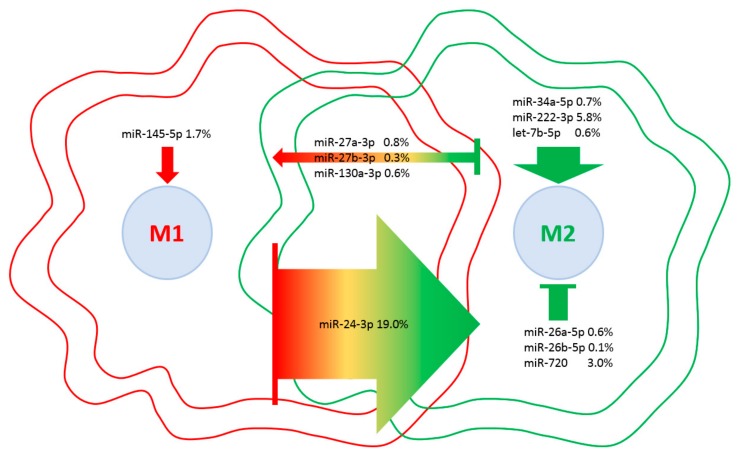
EV-miRNAs involved in M1 or M2 polarization in macrophages. Indication of miRNAs in the first quartile of expression in ASC-EVs and their role in macrophage polarization. miRNAs are divided per category, and the relative amount of their genetic message is shown. Direction of arrows represents the guidance of polarization phenotype. Size of arrows reflects the genetic weight.

Eventually, a database of miRNAs involved in M1 vs. M2 macrophage polarization [48] was scored to weight the influence of embedded EV-miRNAs on macrophage phenotypes (Table 4). Although four miRNAs are involved in M2 with respect to seven controlling M1 phenotype, the presence of miR-24-3p, which is responsible for the M1 to M2 switch, and miR-222-3p, which controls M2 polarization, tip the balance towards a more pronounced influence on M2 phenotype (Figure 8). Consistently, as a global EV fingerprint, 26% of first-quartile miRNA-dependent signals are encompassed by M2-related molecules vs. 7% represented by M1 players. Intriguingly, when IPA CCR5 signaling in the macrophages pathway was searched against the 214 target mRNAs, *JUN*, *FOS* and *MAP2K4* kinases were identified, suggesting an EV-dependent downregulation of the CCR5 cascade, whose inactivation resulted in a switch towards an M2 phenotype [49]. Moreover, to support the influence of EV-miRNAs on monocyte/macrophage homeostasis, miR-16-5p (0.5%), regulating the monocyte to macrophage developmental transition, and miR-34a-5p (0.7%) together with miR-132-3p (0.5%), participating in macrophage maturation, were also found in the frame of the most expressed miRNAs.

## 3. Discussion

In this work, both EV-shuttled miRNAs and soluble factors have been characterized in the secretome of ASCs cultivated in vitro under OA-resembling inflammatory conditions. Several molecules were identified as both cartilage-protective and M1 towards M2 macrophage switchers. Herein presented results provide a strong molecular basis for the promising clinical results observed with ASC-containing products, as MFAT or SVF, in OA settings.

In a murine OA model, injection of MSC secretome, similarly to injection of MSCs, resulted in early pain reduction with a protective effect [15]. Further, both the whole conditioned medium and purified EVs resulted to be effective in cartilage surface restoration and ECM deposition in several in vivo OA defect models [25,27,50,51,52], mainly thorough EV-associated matrix-remodeling enzymes and regulators as a novel means of matrix remodeling in physiological and pathological conditions [53]. In humans, phase I clinical trials demonstrated the safety and preliminary evidence of efficacy and pain reduction by using local injections of autologous ASCs in OA patients [54,55]. Similarly, autologous and minimally manipulated MSC-enriched tissues [56], such as BMAC or MFAT/SVF, showed the same outcomes. Of note, a partially overlapping array of pro-regenerative and anti-inflammatory molecules was recently shown to be secreted from both expanded ASCs and MFAT or lipoaspirates [57], suggesting that signals secreted by MSCs may explain, at least in part, the promising results of MSC-enriched products in OA management.

Although it is quite well-accepted that MSCs act through paracrine mechanisms on resident cells, nevertheless, a thorough characterization of soluble factors and EV content is lacking and would be essential to define MSCs and MSC-enriched products. In this perspective, in the last years, researchers have tried to couple the array of secreted molecules with the observed protective effects in clinical experiences [58,59]. Despite valuable hints, very often, the input datasets had two major flaws. First, secreted factors and EV-miRNAs data were not generated from the same experimental workflow; therefore, technical and/or biological differences were underestimated. Second, for both types of molecules, no inflammatory effects resembling those in the OA joint were considered, being cells cultured in a control medium or under a heavy priming (in the order of ng/mL), which is not close to the concentration of cytokines usually found in synovial fluids (pg/mL). Thus, the present study offers a more homogeneous and comprehensive dataset, being aware that the combination of the three inflammatory cytokines used is a simplified in vitro condition due to the greatly higher number of factors and the wide range of concentration in the joints of OA patients.

Cartilage thinning and degradation are one of the major fingerprints in OA, resulting from an imbalance of the cartilage homeostasis characterized by excessive catabolic activity that overcomes repair capacity. In particular, increased action of the matrix metalloproteinases (MMPs) in combination with reduction of their inhibitors (TIMPs) levels are largely responsible for the earliest osteoarthritic changes [60], suggesting that a restoration of the healthy balance would help in residual cartilage protection and regeneration. Interestingly, in pioneering studies on OA patients, SVF injection was able to stimulate partial regeneration of the articular cartilage, as described by positive changes in the imaging outcomes (MRI or CT) [61,62,63,64,65]. Consistently, in the last years, few clinical trials based on intra-articular injection of autologous ASCs in OA joints reported improvement in pain, function and cartilage volume [66,67]. These effects on cartilage may be, at least partially, ascribed to those protective molecules and miRNAs we found in the ASC secretome. In fact, three out of four of the most abundantly secreted factors (FST, TIMP2 and SERPINE1) are connected with extracellular matrix assembly and homeostasis and are further reported to have a direct influence on cartilage. TIMPs can regulate ECM remodeling and the activities of growth factors and their receptors by inhibiting MMPs [68]. In dogs, *TIMP2* expression is decreased in OA cartilage [69] and synovial fluids [70], whereas, in mice, *TIMP2* depletion leads to accelerated OA [71]. Similar protective effects on cartilage homeostasis were demonstrated in mice for the activin receptor follistatin, whose injection reduced both proteoglycan erosion and synovial macrophage infiltration [72]. Further, Serpin E1, also called PAI-1 or plasminogen activator inhibitor 1, positively correlates with the synthesis of cartilage during pathophysiologic processes [73] and counteracts the activity of urokinase/tissue-type plasminogen activators (uPA/tPA), both elevated in OA cartilage in contrast to reduced serpin E1 [74]. Moreover, the presence of the urokinase plasminogen activator surface receptor (PLAUR) in the secretome (2.4 ng per 10^6^ ASCs) may also contribute to reduce plasminogen (either naturally occurring or secretome-shuttled) activation in plasmin, which can activate MMPs and plays an important role in modulating cartilage function [75]. Furthermore, elafin (PI3) that was found in the secretome at 12 ng per million ASCs may directly inhibit MMP9 [76].

Nevertheless, other abundant molecules, although at lower levels (between 10 and 100 ng with the exception of IGFBP4), have detrimental or ambiguous effects on cartilage homeostasis, making an overall potency prediction less straight-forward. IGFBPs (insuline growth factor-binding proteins) bind chondrocyte-secreted IGF-1, which induces the production of cartilage matrix components and has a protective role in OA [77]. Accordingly, IGF-1 deficiency, partly caused by IGFPBs seizure, caused an increased severity of OA articular cartilage lesions in both rats and guinea pigs [78,79]. Additionally, cathepsin S is a cysteine protease which has been implicated in the pathogenesis of OA and is involved in acute inflammatory conditions of cartilage [80]. Interestingly, Dkk1, a Wnt pathway suppressor, was reported to have dual activity: its supplementation has been shown to protect mice from experimental OA [81], whereas, in rats, the silencing alleviated chondrocyte apoptosis and cartilage destruction [82]. Thus, a stringent control over Wnt-signaling is required to maintain cartilage homeostasis [83]. Further, interleukin 6 is associated with increased levels of MMPs, as well as with the radiographic severity of OA [84]; although, in the secretome, the concomitant presence of its receptor IL6ST may mitigate the detrimental effects. Similarly, the detection of receptors such as IL2RB and TNFRSF1A, with TNFRSF1B/11B and IL1RL1 and CD40 between 1 and 10 ng, may reduce the amount of free OA-related cytokines and chemokines [85]. Thus, the global message of ASC-secreted factors supports the protective and postulated pro-regenerative effects observed in the clinical experience.

This indication is also endorsed by the EV-miRNAs. It clearly emerged that only a sharp definition of the disease context may allow a potency prediction. In fact, each miRNA may target multiple mRNAs which may be absent, weakly expressed or very abundant. In this work, we decided to study the effect of shuttled miRNAs on highly expressed transcripts, in either cartilage or macrophages, being aware that those with low levels might also have important direct regulatory or, through their protein products, active functions. Interestingly, EV-miRNAs appeared to regulate several processes connected with both OA-related transcriptional cascades and migration/cell motility. In particular, the TGFB pathway was demonstrated to play multiple roles in the development, growth and maintenance of articular cartilage [86]. EV-miRNAs appeared to potentially downregulate the TGFBR1/2 branch of the pathway, targeting in addition the downstream effectors SMAD3 and 4. This is of relevance, since TGFBR1 levels and activation correlated with aggrecan and collagen type II levels in human OA cartilage [87]. Nevertheless, a clear picture is again difficult to be drawn, since SMAD5, effector of the alternative ALK1-dependent branch of the TGFB pathway and highly correlating with MMP-13 mRNA levels [87], is also targeted by EV-miRNAs. Thus, the net effect of miRNAs on the protective TGFBR1/Smad2/3 vs. destructive ALK1/Smad1/5 signaling balance may not be evaluable in silico. Under a different point of view, a tight regulation of TGFB may be beneficial, since its supplementation as a therapeutic agent, although leading to prolonged elevation of proteoglycan synthesis and content in articular cartilage, may also induce inflammation, synovial hyperplasia and osteophyte formation [88]. Therefore, ASC-EVs might be an interesting adjuvant therapy to circumvent the undesirable effects of TGFB on cartilage repair. On the contrary, in silico-predicted effects on chondrocyte movements are much sharper, with a strong target on genes involved in cell motility and chemotactic response. Interestingly, in OA cartilage, it has been postulated that chondrocytes may move towards one another and cluster together [89] to secrete inflammatory factors like TNF-α or IL-6. Thus, ASC-EVs could suppress chondrocyte movement and cluster formation observed in pathologic tissues. Eventually, miRNAs effects get even clearer when only those directly involved in cartilage protective or destructive mechanisms are considered (Figure 7). The net weight of shuttled miRNAs is toward protection, especially counteracting inflammatory signals. In particular, miR-24-3p represents 19% of EV-miRNAs and was proposed as therapeutic, since its reduction led to p16INK4a increase and MMP1 secretion, indicating that miR-24-3p is protective against senescence and cartilage catabolism [90]. Additionally, miR-24-3p inhibits chondrocyte apoptosis and helps in reducing OA pathogenic processes in rats [91]. In addition, among the most abundant ones, miR-125b-5p (12.5%) prevents aggrecan loss and miR-222-3p (5.8%) cartilage degradation. Therefore, in agreement with secreted factors, miRNAs have a strong balance towards protective messages on OA cartilage, although few degenerative signals may be identified.

Recently, together with cartilage repair [57], MFAT was also demonstrated to mediate the activity of synovial macrophages [32]. In fact, MFAT released mediators with long-lasting anti-inflammatory properties, unraveling the deep interplay with the OA synovial membrane and/or inflammatory cells presence/homeostasis. Supporting this paradigm, herein reported data showed a clear effect of ASCs factors and miRNAs. In fact, 23 secreted molecules control, at different levels, leukocyte chemotaxis, and 16 are involved with positive regulation. Among the most expressed, the alpha chemokine CXCL10, naturally present in OA synovial fluid, has a pivotal role in the first wave of leukocyte recruitment to the synovium [92]. Of note, CXCL10 was also shown to effectively recruit human subchondral progenitors, suggesting that postulated purely inflammatory molecules may contribute to the migratory effect of synovial fluid, not only on inflammatory cells but also on human subchondral progenitors with healing properties [93]. Similar properties on mesenchymal progenitors were shown for the neutrophil chemoattractant CXCL5, also found at > 10 ng, and T cells-attractant CXCL12, able to enhance the migration of bone marrow MSCs [94,95]. In contrast, CCL2 (> 10 ng) does not recruit human MSCs [96], being a potent soluble mediator involved in macrophage chemotaxis and migration together with other beta chemokines found in ASC secretome as CCL5/8 (> 1 ng), CCL1/3/7/13/21 and MIF (< 1 ng) [96]. Intriguingly, and supporting the effectiveness of the herein proposed in vitro model, MSCs treated with OA synovial fluid showed high expression of CCL2 and IL6 [97], that, together with migration, can direct M2 polarization of monocyte/macrophages [98], suggesting not only an attractant but also a potential anti-inflammatory mechanism. In fact, once monocyte/macrophage accumulate within the inflamed synovial joints as the initial and critical step for OA pathogenesis, their survival, activation and, most importantly, polarization are crucial. In this path, TGFB, CSF1 and IL13 are potent stimulator of the M2 phenotype. They can explain, at least in part, how MSCs facilitate the monocyte to macrophage transition, attenuate already activated M1 macrophages and enhance M2 activation. In this frame, in previous reports, bone marrow MSCs promoted changes in the M1/M2 balance in vitro [99]. This paradigm may also explain the in vitro observation that both ASCs and SVF induced macrophage polarization toward a pro-wound healing M2 type [100]. Therefore, thanks to secreted factors, ASCs in enriched tissues as MFAT or SVF can attenuate M1 macrophages by shifting them to an M2 state, laying the foundations for injury resolution in inflammatory-stimulating environments, such as OA joints.

Intriguingly, in the last years, not only MSCs but also their purified EVs showed similar effects on macrophages with cartilage restoration, pain reduction and a M1 to M2 polarization [27,28,29]. Mining the transcriptome of OA synovia-isolated macrophages, although EV-miRNAs targeted the macrophage-secreted remodeling enzymes, such as MMP1/3/9, they did not directly target and shutdown M1 signals. Albeit, they appeared to induce M2 polarization by interfering with TLR4, which suppresses STAT3, whose predominance over STAT1 increases M2 phenotype [101]. This capacity was emphasized for miRNAs involved in M1 vs. M2 macrophage polarization [48]. Twenty-six percent of miRNA molecules embedded in ASC-EVs are directly involved in M2 regulation (Table 4). Although less in number with respect to M1 miRNAs, the presence of miR-24-3p and miR-222-3p (5.8%) tipped the balance towards a protective phenotype. In particular, miR-24-3p was reported to have a wide field of action in macrophages. First, it is able to mediate the transition between M1 and M2 states through p110δ [102]. Second, it enhanced the ability of IL-4 and IL-13, both found in ASC secretome, to generate a M2 phenotype eventually able to resist M1 re-conversion under inducing stimuli [102]. Third, miR-24-3p is a negative regulator of TLR-mediated pro-inflammatory cytokine production in M1 macrophages [103,104]. Furthermore, miR-222-3p was demonstrated to suppress the expression of SOCS3, an alternative negative regulator of the M2 promoter STAT3 [105]. Remarkably, ingenuity pathway analysis of the CCR5 signaling in macrophages identified JUN, FOS and MAP2K4 kinases as targets, suggesting an EV-dependent downregulation of the CCR5 cascade, whose inactivation resulted in a switch towards an M2 phenotype [49]. Eventually, EV-miRNAs also target genes of the negative regulation of programmed cell death (GO:0043069) and of the apoptotic process (GO:0043066). This means that cell death and turnover might be tightly regulated in macrophages, being thus a further beneficial effect since, in mice, unbiased macrophage depletion did not attenuate the severity of OA but induced systemic inflammation with a massive infiltration of inflammatory T cells and neutrophils into the injured joints [106]. Therefore, not only macrophage polarization but also the maintenance of specific macrophage subtypes, may be necessary to mitigate inflammation and OA.

Thus, being aware that other factors and EV-embedded miRNAs secreted by ASCs were not assayed in the present study and will need further characterization in future experiments, the global message of ASC secretome with a strong contribution of highly secreted TIMP1, TIMP2, PLG and CTSS or miR-24-3p, miR-222-3p and miR-193b-3p supports the protective and postulated pro-regenerative effects observed in the clinical experience and could be potential therapeutic candidates in organ/tissue healing.

## 4. Materials and Methods

### 4.1. Ethics Statement

The research was performed at IRCCS Istituto Ortopedico Galeazzi under Institutional Review Board approval (San Raffaele Hospital Ethics Committee approval in date 8 March 2018, registered under number 6/int/2018). Specimens were collected after obtainment of patient informed consent (CI_REGAIN_adulto_v2) and following the 1964 Helsinki declaration and its later amendments or comparable ethical standards.

### 4.2. Adipose-Derived Mesenchymal Stem Cell (ASCs) Isolation and Expansion

Adipose waste material from four female donors (ASC 1—57 yo, ASC 2—45 yo, ASC 3—54 yo and ASC 4—61 yo) undergoing liposuction was collected. Tissue was digested with type I collagenase (0.075% *w*/*v*, 30 min at 37 °C) (Worthington Biochemical Co, Lakewood, NJ, USA), filtered through a 100 μm cell strainer and centrifuged (1000 × g, 5 min). Pellets were suspended in DMEM + 10% FBS (GE Healthcare, Piscataway, NJ, USA) and penicillin-streptomycin (Life Technology, Carlsbad, CA, USA) and seeded at 5 × 10^3^ cells/cm^2^ in. Cells were cultured at 37 °C, 5% CO_2_ and 95% humidity and analyzed at passage 5 at 90% confluence after five times washed with PBS and eventual addition of FBS-free DMEM for 48 h. Inflammation was performed supplementing FBS-free DMEM with TNFα (5 pg/mL) + IL1β (10 pg/mL) + IFNγ (40 pg/mL) (Peprotech, London, UK), concentrations obtained calculating the median values from [32,33,34,35,36,37,38,39,40]. Conditioned medium (secretome) was collected and centrifuged at 1000× *g* for 15 min at 4 °C, 2000× *g* for 15 min at 4 °C and twice at 4000× *g* for 15 min at 4°C. Clarified supernatant was split and used for EVs analysis and ELISA assays. At time of supernatant collection, cells were detached, counted and viability assessed with Tali image-based cytometer (Thermo Fisher Scientific, Waltham, MA, USA).

### 4.3. ASCs Flow Cytometry Characterization

Flow cytometry was used to score positive or negative MSC (CD44-PE Vio770 clone REA690, CD73-PE clone REA804 and CD90-FITC clone REA897) or hemato/endothelial (CD34-FITC clone AC136, CD31-PerCp Vio700 clone REA730 and CD45-PE Vio770 clone REA747) (Miltenyi Biotec, Bergisch Gladbach, Germany) markers with a CytoFLEX flow cytometer (Beckman Coulter, Fullerton, CA, USA) collecting a minimum of 10,000 events. Doublets and cell aggregates were removed from analysis gating events on FSC-A and FSC-H plots.

### 4.4. ELISA Assays

Concentrations of 200 soluble cytokines, chemokines, receptors and inflammatory and growth factors were determined by Quantibody^®^ Human Cytokine Array 4000 Kit (https://www.raybiotech.com/quantibody-human-cytokine-array-4000/) in the ASC secretomes after, according to the manufacturers’ instructions (RayBiotech, Norcross, GA, USA). Dilutions (1:2) allowed to have absorbance readings within the standard curve values. The amount of each factor in pg/mL was converted into pg/million cells by multiplying the original value for the total volume in ml and, finally, diving by the total number of cells. Values are shown as million cells normalized pg and mean values ± SD calculated.

### 4.5. EVs Isolation and Characterization

Five milliliters of clarified secretomes were 1:2 diluted with PBS and centrifuged at 100,000× *g* for 9 h at 4 °C in a 70Ti rotor (Beckman Coulter, Fullerton, CA, USA), and EV pellets were processed as follows:

(i) Flow cytometry: Before ultracentrifugation, secretomes were additioned with 10 µM CFSE (Sigma-Aldrich) and incubated for 1 h at 37 °C. After ultracentrifugation, as previously described, labelled EVs were suspended in 50 µl PBS per 5 mL of processed medium. Particles were 1:10,000 diluted in PBS and 100 µl stained with anti-5 μL CD9-APC clone HI9a, anti-CD63-APC clone H5C6 and CD81-APC clone 5A6 (Biolegend, San Diego, CA, USA) for 30 min at 4 °C in the dark. Antibodies were used individually. Events collection was performed with a CytoFLEX flow cytometer collecting a minimum of 30,000 events. A reference bead mix (Biocytex, Marseille, France) composed of a FITC fluorescent mixture of spheres (100 nm, 300 nm, 500 nm and 900 nm) was used to set up the flow cytometer. Gains were: FSC = 106, SSC = 61, FITC = 272, PE = 116 and PC7 = 371. FITC threshold was set at 500 to include 100 nm beads and some smaller debris in the FITC channel.
(ii) Transmission electron microscopy: Five microliters of PBS-suspended EVs were absorbed on Formvar carbon-coated grids for 10 min. Filter paper was used to blot drops. Negative stain was performed with 2% uranyl acetate aqueous suspension for 10 min, and excess was removed by filter paper before drying the grid at RT. Samples were examined with a TALOS L120C transmission electron microscope (Thermo Fisher Scientific, Waltham, MA, USA) at 120 kV.
(iii) Nanoparticle tracking analysis (NTA): Purified EVs in PBS (1:100 diluted) were visualized by Nanosight LM10-HS system (NanoSight Ltd., Amesbury, UK). Three recordings of 30 s were performed for each sample. NTA software was used to analyze the data and provided both the concentration measurements and the high-resolution particle size distribution profiles.

### 4.6. Screening of EV-Embedded miRNA Expression

After ultracentrifugation, Trizol was used to dissolve EV pellets, and miRNeasy Kit and RNeasy CleanUp Kit were used sequentially to obtain RNA enriched in small molecules < 200 nt (Qiagen, Hilden, Germany). During extraction, 6 pg of a nonhuman synthetic miRNA (arabidopsis thaliana ath-miR-159a) were added to each sample as a spike-in to monitor the technical variability during both the isolation and the following reactions. The spike-in was further used to equalize A and B panels of the OpenArray^®^ platform (Life Technologies). Briefly, cDNAs were prepared by standard reverse transcription (RT), and preamplification was performed with A and B independent kits, followed by real-time RT-PCR analysis with the QuantStudio™ 12 K Flex OpenArray^®^ Platform (QS12KFlex), as previously described [107]. To process miRNA expression data from the A and B miRNA panels, together covering 754 human miRNA sequences from the Sanger miRBase v21 Gene, the Expression Suite Software (Life Technologies) was used. C_RT_ of 28 was considered as a burden for the absence or presence of amplification. The global mean was selected as a normalization method [108] and was calculated from miRNAs positively amplified in all samples. Normalized miRNA expression was determined using the relative quantification 2−Δ^C^_RT_. Values are shown as normalized Ct and mean values ± SD calculated.

### 4.7. Pathway Analysis

Identification of functional annotations:

(i) Protein: Secretome-identified factors were subjected to functional enrichment analysis to provide insight into the functional associations of these protein subsets. The analysis was performed using Gorilla tool (http://cbl-gorilla.cs.technion.ac.il/) with two unranked lists of genes (target and background lists) running mode and 200 factors of the ELISA array as background [109,110]. The list of proteins was also submitted to the PANTHER web interface (http://www.pantherdb.org/) to identify proteins belonging to the same functional classifications, following default settings [111].
(ii) miRNA: The predicted miRNA targets were annotated into functional BP using the microRNA Target Filter tool in ingenuity pathway analysis (IPA; Ingenuity^®^ Systems, http://www.ingenuity.com). Filters were: confidence “experimentally observed”, with all sources available in the IPA database (TarBase, miRecords, TargetScan Human and Ingenuity Expert Findings). To obtain the list of all experimentally validated mRNA targets, no disease filter was set.

### 4.8. Hierarchical Clustering

Heat map plots were generated, scoring factors or miRNAs with ClustVis package (https://biit.cs.ut.ee/clustvis/) [112]. Clustering options were: distance for rows—correlation, method for rows—average and tree ordering for rows—tightest cluster first. Log_2_ [(pg) factors per 10^6^ cells] or miRNA C_RT_ after normalization values were used.

### 4.9. Statistical Analyses

In each experiment, four independent ASCs were analyzed. Only proteins or miRNAs present and quantified in all conditions were considered as positively identified. GraphPad Prism software (GraphPad, San Diego, CA, USA) was used for statistical analysis. Kolmogorov–Smirnov normality test assayed normal data distribution. Grubbs’ test identified outliers. The level of significance was set with a minimum *p*-value < 0.05. Data was presented as mean ± SD (Appendix A).

## 5. Conclusions

Secreted factors and EV-embedded miRNAs may explain the promising results observed for both expanded ASCs and ASC-enriched products as SVF or MFAT. Both molecules and miRNAs may profoundly alter cartilage homeostasis and macrophage polarization, supporting the protective and pro-regenerative effects observed in the clinical experience. We think that this report shed light on some mechanisms that mesenchymal stem cells, alone or in their niche, activate to heal a diseased joint. Future studies will be needed, especially under the light of new transcriptomes being daily released for the other cellular players, as the identification of the targets appeared as a mandatory sieve to deeply understand cellular and molecular interactions.

## Figures and Tables

**Table 1:** pg per 10^6^ adipose-derived MSCs (ASCs) in 48 h.

FACTOR	ASC 1	ASC 2	ASC 3	ASC 4	MEAN	SD	DESCRIPTION
FST	1,492,673	1,081,479	570,416	1,004,932	1,037,375	377,865	Follistatin
TIMP2	167,790	162,350	198,481	256,519	196,285	43,191	Metalloproteinase inhibitor 2
IGFBP4	223,536	276,754	145,198	84,223	182,428	84,887	Insulin-like growth factor-binding protein 4
SERPINE1	109,335	103,661	98,032	119,475	10,7626	9148	Plasminogen activator inhibitor 1
IGFBP6	86,610	83,502	77,842	94,332	85,572	6877	Insulin-like growth factor-binding protein 6
IL6ST	68,647	68,361	36,585	50,182	55,944	15,530	Interleukin-6 receptor subunit beta
CTSS	52,275	50,380	40,226	64,808	51,922	10,089	Cathepsin S
IL6	43,025	30,591	40,435	82,769	49,205	23,008	Interleukin-6
TIMP1	34,985	27,024	33,210	41,839	34,265	6094	Metalloproteinase inhibitor 1
ICAM1	28,715	21,857	22,377	33,311	26,565	5472	Intercellular adhesion molecule 1
CXCL10	28,629	21,386	16,094	38,665	26,193	9774	C-X-C motif chemokine 10
IL2RB	28,885	20,962	18,101	27,057	23,751	5066	Interleukin-2 receptor subunit beta
TNFRSF1A	15,652	21,277	21,328	13,791	18,012	3875	Tumor necrosis factor receptor superfamily member 1A
CCL2	16,286	13,411	15,771	19,076	16,136	2325	C-C motif chemokine 2
PLG	17,218	14,055	14,936	17,793	16,001	1790	Plasminogen
CXCL5	15,226	5628	5204	36,614	15,668	14,711	C-X-C motif chemokine 5
DKK1	17,275	12,422	8703	9540	11,985	3870	Dickkopf-related protein 1
PI3	18,953	3218	6567	20,207	12,236	8605	Elafin (Peptidase inhibitor 3)
CCL5	15,611	4502	4407	11,970	9122	5591	C-C motif chemokine 5
FLT1	5604	4854	13,916	10,834	8802	4324	Vascular endothelial growth factor receptor 1
IGFBP3	4898	11,609	5145	11,349	8250	3731	Insulin-like growth factor-binding protein 3
ANG	4612	8990	5615	2158	5344	2831	Angiogenin
SHH	6957	5751	3445	5437	5398	1457	Sonic hedgehog protein
GDF15	5128	6376	4534	4167	5051	968	Growth/differentiation factor 15
CXCL9	4391	2472	1272	10,677	4703	4185	C-X-C motif chemokine 9
CSF1	4488	3510	3624	2797	3604	693	Macrophage colony-stimulating factor 1
CXCL1	3334	3781	2516	4276	3477	747	Growth-regulated alpha protein
IL1RL1	5212	1440	3249	3991	3473	1579	Interleukin-1 receptor-like 1
TNFRSF11B	4378	1861	4234	2934	3352	1187	Tumor necrosis factor receptor superfamily member 11B
CXCL8	2521	3046	2715	3839	3030	581	Interleukin-8
TNFRSF1B	2277	3327	1014	5025	2911	1697	Tumor necrosis factor receptor superfamily member 1B
VEGFA	1956	2378	1738	5600	2918	1808	Vascular endothelial growth factor A
PLAUR	2404	3497	2581	1114	2399	981	Urokinase plasminogen activator surface receptor
CSF3	1989	897	770	6605	2565	2748	Granulocyte colony-stimulating factor
CCL8	1107	2046	1098	3351	1900	1064	C-C motif chemokine 8
CCL4	1851	1055	1312	2689	1727	722	C-C motif chemokine 4
CD40	1730	1681	841	1380	1408	408	Tumor necrosis factor receptor superfamily member 5
CD14	1467	2114	1175	555	1328	648	Monocyte differentiation antigen CD14
EGFR	1006	1602	792	1938	1335	529	Epidermal growth factor receptor
AXL	958	2294	1183	277	1178	838	Tyrosine-protein kinase receptor UFO
TREM1	961	1063	709	1549	1071	352	Triggering receptor expressed on myeloid cells 1
TYRO3	2334	123	1155	494	1026	971	Tyrosine-protein kinase receptor TYRO3
HGF	823	832	1407	778	960	299	Hepatocyte growth factor
LIF	676	834	1014	1295	954	265	Leukemia inhibitory factor
CXCL16	514	1256	539	993	825	362	C-X-C motif chemokine 16
CXCL6	1144	546	41	1123	714	527	C-X-C motif chemokine 6
NRCAM	574	723	50	1424	693	567	Neuronal cell adhesion molecule
CCL21	731	1164	384	289	642	397	C-C motif chemokine 21
TGFB1	769	793	630	363	639	197	Human TGF-beta 1
SPP1	189	434	635	917	544	308	Osteopontin
TNFRSF10D	637	103	330	1033	526	403	Tumor necrosis factor receptor superfamily member 10D
FAS	608	399	540	203	438	179	Tumor necrosis factor receptor superfamily member 6
FGF7	617	308	575	208	427	200	Fibroblast growth factor 7
SIGLEC5	456	545	288	446	434	107	Sialic acid-binding Ig-like lectin 5
IL23A	441	425	166	655	422	200	Interleukin-23 subunit alpha
IL17B	532	453	438	156	395	164	Interleukin-17B
MIF	376	285	446	479	396	86	Macrophage migration inhibitory factor
CCL1	88	112	73	1214	371	562	C-C motif chemokine 1
CD40LG	80	268	220	588	289	215	CD40 ligand
CCL13	166	155	297	490	277	156	C-C motif chemokine 13
VEGFC	568	215	160	23	241	232	Vascular endothelial growth factor C
CCL11	159	103	416	294	243	141	Eotaxin
IL1B	271	179	187	294	233	58	Interleukin-1 beta
TNF	81	123	151	590	236	237	Tumor necrosis factor
ENG	162	128	254	179	181	53	Endoglin
TNFRSF21	62	199	76	192	132	73	Tumor necrosis factor receptor superfamily member 21
CCL7	58	72	54	362	137	150	C-C motif chemokine 7
CCL3	90	19	25	251	96	108	C-C motif chemokine 3
IL2RA	108	74	72	58	78	21	Interleukin-2 receptor subunit alpha
AGRP	17	125	16	113	68	59	Agouti-related protein
IL1A	85	25	26	115	63	45	Interleukin-1 alpha
CXCL12	71	92	19	28	52	35	Stromal cell-derived factor 1
IL17A	87	1	34	78	50	40	Interleukin-17A
PGF	25	8	20	100	38	42	Placenta growth factor
IL13	18	9	11	25	16	7	Interleukin-13

Colors represent factor amount, red for high and blue for low.

**Table 2:** Enriched gene ontology (GO) terms for secreted factors.

Factor Amount	GO TERM	Description	*p*-Value	Factors
> 10 ng/10^6^ ASC	GO:0022411	cellular component disassembly	3.74 × 10^−6^	TIMP1, TIMP2, DKK1, PLG and CTSS
	GO:0022617	extracellular matrix disassembly	5.13 × 10^−5^	TIMP1, TIMP2, PLG and CTSS
< 10 ng/10^6^ ASC	GO:0002791	regulation of peptide secretion	8.05 × 10^−4^	IL17A, ANG, VEGFC, TGFB1, CCL5, IL13, IL1RL1, MIF, CD40LG, IL1B, CD40, CCL1, IL1A, CCL3, TNFRSF21, TNF, EGFR, CD14 and TNFRSF1B
	GO:0050708	regulation of protein secretion	8.05 × 10^−4^	IL17A, ANG, VEGFC, TGFB1, CCL5, IL13, IL1RL1, MIF, CD40LG, CCL1, CD40, IL1B, IL1A, CCL3, TNFRSF21, TNF, EGFR, CD14 and TNFRSF1B
	GO:0022603	regulation of anatomical structure morphogenesis	8.07 × 10^−4^	SHH, FLT1, CXCL9, GDF15, LIF, PGF, CXCL12, SPP1, FGF7, ENG, CSF1, VEGFA, CCL13, VEGFC, CCL11, TGFB1, CCL8, CCL7, IL1B, CD40, IL1A, CCL3, HGF, TNF, IL8, TNFRSF1B, NRCAM and TNFSRSF11B
	GO:0043903	regulation of symbiosis	8.22 × 10^−4^	TNF, IL8, CCL5, CCL4, CCL8, CXCL6 and CCL3
	GO:0070201	regulation of protein localization	9.16 × 10^-4^	IL17A, ANG, VEGFC, TGFB1, SHH, CCL5, IL13, IL1RL1, MIF, CD40LG, CCL1, IL1B, CD40, IL1A, CCL3, TNFRSF21, TNF, EGFR, CD14 and TNFRSF1B
	GO:0051223	regulation of protein transport	9.16 × 10^−4^	IL17A, ANG, VEGFC, TGFB1, SHH, CCL5, IL13, IL1RL1, MIF, CD40LG, IL1B, CD40, CCL1, IL1A, CCL3, TNFRSF21, TNF, EGFR, CD14 and TNFRSF1B
	GO:0090087	regulation of peptide transport	9.16 × 10^−4^	IL17A, ANG, VEGFC, TGFB1, SHH, CCL5, IL13, IL1RL1, MIF, CD40LG, CCL1, CD40, IL1B, IL1A, CCL3, TNFRSF21, TNF, EGFR, CD14 and TNFRSF1B
	GO:0006954	inflammatory response	9.98 × 10^−4^	IL17A, IL17B, CXCL9, IL13, MIF, SPP1, IL23A, AXL, CD14, CSF1, CCL13, CCL11, TGFB1, CCL5, IL2RA, CCL4, CCL8, CCL7, CD40LG, TREM1, IL1B, CD40, CCL1, IL1A, CCL3, TNF, CXCL1, IL8, CXCL6, TNFRSF1B and CCL21

**Table 3:** First-quartile extracellular vesicle (EV)-miRNAs involved in osteoarthritis (OA) pathogenesis.

Protective				
miRNA	% EV-genetic weight	Target	Tissue	Role
hsa-miR-24-3p	19.0	*P16INK4A*	Cartilage	Regulates senescence
hsa-miR-222-3p	5.8	*HDAC4*	Cartilage	Prevents cartilage degradation
hsa-miR-193b-3p	5.0	*TGFB2-3/MMP19*	Cartilage	Regulates inflammation
hsa-miR-92a-3p	1.5	*ADAMTS4-5/HDAC2*	Cartilage	Anti-catabolic
hsa-miR-29a-3p	1.1	*VEGF*	Synovium	Inhibits synovial remodeling
hsa-miR-199a-3p	0.9	*COX2*	Cartilage	Anti-catabolic
hsa-miR-27a-3p	0.8	*FSTL1*	Synovium	Prevents synoviocyte migration
hsa-miR-26a-5p	0.6	*iNOS*	Cartilage	Cartilage homeostasis
hsa-miR-130a-3p	0.6	*TNFa*	Cartilage	Anti-inflammatory
hsa-miR-320a-3p	0.5	*BMI1/RUNX2/MMP13*	Cartilage	Chondrogenic
hsa-miR-152-3p	0.5	*DNMT*	Synovium	Prevents synoviocyte migration
hsa-miR-17-5p	0.5	*SQSTM1*	Cartilage	Induces Autophagy
hsa-miR-27b-3p	0.3	*MMP13/LEPTIN*	Cartilage	Anti-catabolic
hsa-miR-210-3p	0.2	*DR6/HIF3a*	Cartilage	Anti-apoptotic/promotes ECM
hsa-miR-30a-5p	0.2	*ADAMTS5*	Cartilage	Cartilage homeostasis
hsa-miR-26b-5p	0.1	*KPNA3*	Cartilage	Promotes NF-kB pathway
hsa-miR-149-5p	0.1	*TNFa*	Cartilage	Anti-inflammatory
**Total**	**37.9**			
**Destructive**				
**miRNA**	**% EV-genetic weight**	**Target**	**Tissue**	**Role**
hsa-miR-21-5p	4.7	*GDF5*	Cartilage	Chondrogenesis inhibitor
hsa-miR-30b-5p	2.4	*BECN1/ATG5*	Cartilage	ECM degradation
hsa-miR-19b-3p	1.4	*nd*	Synovium	Induces NF-kB signaling
hsa-miR-34a-5p	0.7	*SIRT1*	Cartilage	Apoptotic
hsa-miR-16-5p	0.4	*SMAD3*	Cartilage	Cartilage degradation
hsa-miR-138-5p	0.2	*FOXC1*	Cartilage	Promotes cartilage degradation
hsa-miR-483-5p	0.1	*MATN3/TIMP2*	Cartilage	ECM degradation
**Total**	**9.8**			
**Protective/Destructive**				
**miRNA**	**% EV-genetic weight**	**Target**	**Tissue**	**Role**
hsa-miR-125b-5p	12.6	*ADAMTS4/* *SYVN1*	Cartilage/Synovium	Prevents aggrecan loss/Pro-apoptotic
hsa-miR-221-3p	4.7	*SDF1/* *nd*	Cartilage	Prevents ECM degradation/Pro-inflammatory
hsa-miR-145-5p	1.7	*TNFRSF11B/* *SOX9-SMAD3*	Cartilage	Chondrocyte proliferation/Cartilage degradation
hsa-miR-365-3p	0.2	*HIF2a/* *HDAC4*	Cartilage	Prevents ECM loss/Pro-inflammatory
**Total**	**19.2**			

**Table 4:** First-quartile EV-miRNAs involved in macrophage polarization.

M1 Polarization		
miRNAs	% EV-Genetic Weight	Regulation of Macrophage Phenotype
hsa-miR-145-5p	1.7	Promotes M1
hsa-miR-27a-3p	0.8	Promotes M1, suppresses M2
hsa-miR-27b-3p	0.3	Promotes M1, suppresses M2
hsa-miR-130a-3p	0.6	Promotes M1, suppresses M2
hsa-miR-26a-5p	0.6	Suppresses M2
hsa-miR-26b-5p	0.1	Suppresses M2
hsa-miR-720	3.0	Suppresses M2
**Total**	**7.2**	
**M2 Polarization**		
**miRNAs**	**% EV-Genetic Weight**	**Regulation of Macrophage Phenotype**
hsa-miR-34a-5p	0.7	Promotes M2
hsa-miR-222-3p	5.8	Promotes M2
hsa-let-7b-5p	0.6	Promotes M2
hsa-miR-24-3p	19.0	Promotes M2, suppresses M1
**Total**	**26.1**	

## Supplementary Materials

Supplementary materials can be found at https://www.mdpi.com/1422-0067/21/5/1582/s1.

## Abbreviations

OA	Osteoarthritis
MSC	Mesenchymal Stem Cells
EV	Extracellular Vesicles
miRNA	MicroRNA
HA	Hyaluronic Acid
ECM	Extracellular Matrix
GO	Gene Ontology
BP	Biological Process

## Appendix A

Raw data can be found at OSF Repository, https://osf.io/kjgxq/.
