# Supplementary material for: Secreted Factors and EV-miRNAs Orchestrate the Healing Capacity of Adipose Mesenchymal Stem Cells for the Treatment of Knee Osteoarthritis
Source: Int J Mol Sci. 2020 Feb 26;21(5):1582. doi: 10.3390/ijms21051582 (PMC7084308; doi:10.3390/ijms21051582)
Supplement: Supplementary file 1 [file ijms-21-01582-s001.zip › Supplementary Table 4_IJMS.docx]

Supplementary Table 4. Target mRNAs for 65 EV-miRNAs in the first quartile of expression that were filtered through highly expressed OA-synovial macrophages genes

| ACP2 | CSF1 | INSIG1 | PDCD4 | STK40 |
| --- | --- | --- | --- | --- |
| ACTR1A | CSNK1D | ISCU | PDCD6IP | STX7 |
| ADAMTS1 | CTNND1 | ITGA5 | PELI1 | SWAP70 |
| ADPGK | CXCL8 | JAG1 | PHB | SYPL1 |
| AHNAK | CYP1B1 | JAK1 | PLAU | TGFBR1 |
| ALOX5 | DAD1 | JUN | PMAIP1 | TGFBR2 |
| ANPEP | DDIT4 | JUNB | PNP | TIMP3 |
| AP2M1 | DNAJB4 | KCNN4 | PPIF | TLR4 |
| APP | DUSP2 | KLF4 | PRDM1 | TLR7 |
| ARAF | EGR2 | LAMP2 | PRDX6 | TMCO1 |
| ARHGDIA | EIF4E | LAMTOR3 | PRPF40A | TMED10 |
| ARID4B | EIF4G2 | LAMTOR5 | PTGS2 | TMED2 |
| ARL2 | F11R | LIPA | PTPN1 | TMEM109 |
| ARPC3 | FGFR1 | LRRC8C | PXN | TMEM43 |
| ATF4 | FN1 | LRRFIP1 | RAB21 | TMEM59 |
| ATF6 | FNDC3A | MAFB | RAF1 | TMEM87A |
| ATP6V1F | FNDC3B | MAN1A1 | RAP1B | TNF |
| BECN1 | FOS | MAP2K1 | RB1 | TNFAIP3 |
| BMP2K | FOXO1 | MAP4K4 | RBMS1 | TNFRSF10B |
| BNIP2 | FOXO3 | MAPRE1 | RDH10 | TP53I11 |
| BNIP3L | FOXP1 | MAPRE2 | RHOB | TPI1 |
| BSG | FURIN | MARCKS | RHOC | TPM3 |
| BTG2 | GAS7 | MAT2A | RHOG | TPPP3 |
| C11orf58 | GMFB | MAX | RTN4 | TSPAN3 |
| CALCOCO2 | GNAI2 | MBNL1 | RUNX1 | TUBB2A |
| CALU | GRB2 | MCL1 | scD | UAP1 |
| CAPG | GTF2H1 | MEF2D | SCYL1 | UBE2S |
| CAPRIN1 | H3F3A | MGAT4A | SGK3 | UCP2 |
| CASP3 | HBP1 | MMP3 | SGPL1 | UGDH |
| CCND1 | HGS | MMP9 | SIRT1 | UGP2 |
| CD276 | HIF1A | MPDU1 | SKAP2 | VEGFA |
| CD44 | HIPK3 | MTPN | SLC16A10 | VIM |
| CD47 | HMGA1 | MYC | SMARCA5 | VTI1B |
| CDC42 | HMOX1 | MYD88 | SMC1A | WIPF1 |
| CDK4 | HNRNPM | MYLIP | SMOX | XBP1 |
| CDKN1A | HSP90B1 | NASP | SNAP23 | ZFP36L1 |
| CHMP2A | ICAM1 | NCEH1 | SOD2 | ZNF622 |
| CHORDC1 | ID2 | NCL | SPARC | ZYX |
| COL1A1 | IFI16 | NDUFA4 | SPI1 |  |
| COL1A2 | IFRD1 | NOTCH2 | SQSTM1 |  |
| COL3A1 | IL10 | NR4A2 | SRPRA |  |
| COMMD9 | IL1RN | NUCB1 | SRSF10 |  |
| CREBL2 | IL6 | NUFIP2 | ST14 |  |
| CSDE1 | IL6R | OGT | STAT3 |  |
