# Supplementary material for: Secreted Factors and EV-miRNAs Orchestrate the Healing Capacity of Adipose Mesenchymal Stem Cells for the Treatment of Knee Osteoarthritis
Source: Int J Mol Sci. 2020 Feb 26;21(5):1582. doi: 10.3390/ijms21051582 (PMC7084308; doi:10.3390/ijms21051582)
Supplement: Supplementary file 1 [file ijms-21-01582-s001.zip › Supplementary Table 1_IJMS.docx]

| Supplementary Table 1. Normalized C_RT_ values for EV-embedded miRNas | | | | | | |
| --- | --- | --- | --- | --- | --- | --- |
| Target Name | ASC 1 | ASC 2 | ASC 3 | ASC 4 | MEAN | SD |
| hsa-miR-24-3p | 9.22 | 9.13 | 9.40 | 9.10 | 9.21 | 0.13 |
| hsa-miR-125b-5p | 9.75 | 10.25 | 9.91 | 9.33 | 9.81 | 0.38 |
| hsa-miR-222-3p | 10.80 | 10.84 | 11.12 | 10.95 | 10.93 | 0.15 |
| hsa-miR-193b-3p | 11.27 | 10.85 | 11.37 | 11.08 | 11.14 | 0.23 |
| hsa-miR-221-3p | 11.50 | 10.79 | 11.28 | 11.30 | 11.22 | 0.30 |
| hsa-miR-21-5p | 10.81 | 11.64 | 11.26 | 11.23 | 11.24 | 0.34 |
| hsa-miR-100-5p | 11.64 | 11.92 | 12.07 | 11.32 | 11.74 | 0.33 |
| hsa-miR-30c-5p | 11.55 | 11.75 | 11.70 | 12.09 | 11.77 | 0.23 |
| hsa-miR-720 | 12.92 | 11.79 | 11.36 | 11.36 | 11.86 | 0.74 |
| hsa-miR-99a-5p | 11.73 | 12.07 | 12.07 | 11.59 | 11.86 | 0.24 |
| hsa-miR-30b-5p | 11.88 | 12.15 | 12.31 | 12.45 | 12.20 | 0.25 |
| hsa-miR-191-5p | 12.67 | 12.47 | 12.83 | 12.63 | 12.65 | 0.15 |
| hsa-miR-145-5p | 12.54 | 13.19 | 12.07 | 12.93 | 12.68 | 0.49 |
| hsa-miR-92a-3p | 13.21 | 12.65 | 12.68 | 13.12 | 12.91 | 0.29 |
| hsa-miR-31-5p | 12.94 | 13.45 | 12.92 | 12.50 | 12.96 | 0.39 |
| hsa-miR-19b-3p | 12.87 | 12.74 | 12.94 | 13.55 | 13.02 | 0.36 |
| hsa-miR-20a-5p | 13.15 | 12.79 | 12.88 | 13.35 | 13.04 | 0.26 |
| hsa-miR-99b-5p | 12.87 | 13.44 | 13.28 | 12.78 | 13.09 | 0.32 |
| hsa-miR-29a-3p | 13.38 | 12.62 | 13.65 | 13.63 | 13.32 | 0.48 |
| hsa-miR-574-3p | 13.62 | 13.23 | 13.65 | 13.46 | 13.49 | 0.20 |
| hsa-miR-214-3p | 13.55 | 13.58 | 13.46 | 13.40 | 13.50 | 0.08 |
| hsa-miR-199a-3p | 13.35 | 13.86 | 13.72 | 13.52 | 13.61 | 0.23 |
| hsa-miR-27a-3p | 13.31 | 14.10 | 13.90 | 13.66 | 13.74 | 0.34 |
| hsa-miR-218-5p | 13.60 | 13.62 | 13.52 | 14.49 | 13.81 | 0.46 |
| hsa-miR-127-3p | 13.68 | 14.16 | 13.92 | 13.96 | 13.93 | 0.20 |
| hsa-miR-382-5p | 13.80 | 13.68 | 14.14 | 14.23 | 13.96 | 0.26 |
| hsa-miR-34a-5p | 13.76 | 14.54 | 14.20 | 13.60 | 14.03 | 0.43 |
| hsa-miR-224-5p | 13.80 | 14.00 | 13.83 | 15.11 | 14.19 | 0.62 |
| hsa-miR-26a-5p | 13.96 | 14.89 | 14.18 | 13.72 | 14.19 | 0.51 |
| hsa-miR-130a-3p | 14.08 | 14.11 | 14.26 | 14.34 | 14.20 | 0.12 |
| hsa-let-7b-5p | 14.37 | 14.79 | 14.44 | 13.55 | 14.29 | 0.53 |
| hsa-miR-328-3p | 14.23 | 14.39 | 14.48 | 14.05 | 14.29 | 0.19 |
| hsa-miR-320a-3p | 14.64 | 14.17 | 14.49 | 14.22 | 14.38 | 0.22 |
| hsa-miR-152-3p | 14.25 | 14.58 | 14.49 | 14.30 | 14.40 | 0.15 |
| hsa-miR-29c-3p | 14.39 | 14.05 | 14.68 | 14.60 | 14.43 | 0.28 |
| hsa-miR-132-3p | 14.68 | 14.67 | 14.51 | 14.02 | 14.47 | 0.31 |
| hsa-miR-484 | 14.50 | 13.92 | 14.67 | 14.88 | 14.49 | 0.41 |
| hsa-miR-17-5p | 14.74 | 13.92 | 14.48 | 14.89 | 14.51 | 0.43 |
| hsa-miR-106a-5p | 14.84 | 13.98 | 14.42 | 15.07 | 14.58 | 0.48 |
| hsa-miR-197-3p | 14.47 | 14.51 | 14.95 | 14.55 | 14.62 | 0.22 |
| hsa-miR-16-5p | 14.58 | 14.72 | 14.78 | 14.89 | 14.74 | 0.13 |
| hsa-miR-106b-5p | 14.84 | 15.19 | 15.24 | 15.53 | 15.20 | 0.28 |
| hsa-miR-27b-3p | 14.56 | 15.55 | 15.51 | 15.21 | 15.21 | 0.46 |
| hsa-miR-331-3p | 15.01 | 15.86 | 15.39 | 15.23 | 15.37 | 0.36 |
| hsa-miR-376a-3p | 15.00 | 15.30 | 15.42 | 16.14 | 15.46 | 0.48 |
| hsa-let-7a-5p | 15.61 | 16.58 | 15.66 | 14.40 | 15.56 | 0.89 |
| hsa-miR-601 | 15.80 | 15.17 | 17.68 | 13.69 | 15.58 | 1.65 |
| hsa-miR-210-3p | 16.04 | 15.71 | 15.44 | 15.23 | 15.60 | 0.35 |
| hsa-miR-30a-5p | 15.32 | 16.04 | 15.91 | 15.16 | 15.61 | 0.43 |
| hsa-miR-10a-5p | 15.43 | 16.11 | 15.50 | 15.58 | 15.65 | 0.31 |
| hsa-let-7c-5p | 15.52 | 15.73 | 15.77 | 15.61 | 15.65 | 0.11 |
| hsa-miR-138-5p | 15.73 | 16.44 | 16.34 | 14.91 | 15.85 | 0.70 |
| hsa-miR-376c-3p | 15.75 | 15.79 | 16.24 | 16.31 | 16.02 | 0.30 |
| hsa-miR-22-3p | 15.78 | 16.17 | 16.08 | 16.21 | 16.06 | 0.19 |
| hsa-miR-409-3p | 16.08 | 15.90 | 16.37 | 15.91 | 16.06 | 0.22 |
| hsa-miR-365-3p | 16.23 | 16.09 | 16.04 | 16.11 | 16.12 | 0.08 |
| hsa-miR-193a-5p | 16.64 | 15.75 | 16.01 | 16.19 | 16.15 | 0.37 |
| hsa-miR-25-3p | 16.16 | 15.67 | 16.09 | 16.71 | 16.16 | 0.43 |
| hsa-miR-31-3p | 16.09 | 16.63 | 16.53 | 15.58 | 16.21 | 0.48 |
| hsa-miR-26b-5p | 15.97 | 16.79 | 16.39 | 15.96 | 16.28 | 0.40 |
| hsa-miR-28-5p | 16.23 | 16.67 | 16.28 | 16.13 | 16.33 | 0.24 |
| hsa-miR-296-5p | 16.37 | 16.48 | 16.13 | 16.60 | 16.39 | 0.20 |
| hsa-miR-28-3p | 16.44 | 16.21 | 16.69 | 16.77 | 16.53 | 0.26 |
| hsa-miR-149-5p | 16.38 | 16.35 | 16.42 | 16.98 | 16.53 | 0.30 |
| hsa-miR-483-5p | 16.34 | 16.59 | 16.47 | 16.77 | 16.54 | 0.18 |
| hsa-miR-1260a | 17.92 | 16.34 | 15.95 | 16.17 | 16.60 | 0.90 |
| hsa-miR-195-5p | 16.15 | 16.86 | 16.66 | 16.72 | 16.60 | 0.31 |
| hsa-miR-370-3p | 16.52 | 16.64 | 16.72 | 16.98 | 16.71 | 0.20 |
| hsa-miR-15b-5p | 16.42 | 16.68 | 16.65 | 17.15 | 16.73 | 0.31 |
| hsa-miR-181a-5p | 16.76 | 18.03 | 16.51 | 15.72 | 16.75 | 0.96 |
| hsa-miR-143-3p | 16.24 | 17.80 | 16.12 | 16.88 | 16.76 | 0.77 |
| hsa-miR-335-5p | 17.35 | 17.20 | 16.04 | 16.66 | 16.81 | 0.59 |
| hsa-miR-23a-3p | 16.85 | 16.79 | 17.08 | 16.66 | 16.84 | 0.17 |
| hsa-miR-361-5p | 16.78 | 17.06 | 16.93 | 17.04 | 16.95 | 0.13 |
| hsa-miR-886-3p | 17.32 | 16.81 | 16.24 | 17.49 | 16.96 | 0.56 |
| hsa-miR-148a-3p | 16.88 | 16.86 | 16.68 | 17.65 | 17.02 | 0.43 |
| hsa-miR-532-5p | 17.08 | 17.01 | 17.33 | 16.97 | 17.10 | 0.16 |
| hsa-miR-29b-3p | 17.24 | 17.76 | 17.42 | 16.29 | 17.18 | 0.63 |
| hsa-miR-339-5p | 17.27 | 16.77 | 17.24 | 17.73 | 17.25 | 0.39 |
| hsa-miR-130b-3p | 17.27 | 16.78 | 17.24 | 18.09 | 17.35 | 0.54 |
| hsa-miR-324-5p | 16.99 | 17.85 | 17.43 | 17.21 | 17.37 | 0.37 |
| hsa-miR-660-5p | 17.38 | 17.50 | 17.46 | 17.35 | 17.42 | 0.07 |
| mmu-miR-93-5p | 17.66 | 16.86 | 17.48 | 17.83 | 17.46 | 0.42 |
| hsa-miR-342-3p | 17.57 | 17.32 | 17.61 | 17.46 | 17.49 | 0.13 |
| hsa-miR-663b | 18.58 | 16.88 | 17.06 | 18.16 | 17.67 | 0.83 |
| hsa-miR-374a-5p | 17.53 | 18.80 | 17.50 | 17.09 | 17.73 | 0.74 |
| mmu-miR-140-5p | 17.71 | 18.00 | 17.64 | 17.99 | 17.84 | 0.19 |
| hsa-miR-886-5p | 18.48 | 17.79 | 17.15 | 18.02 | 17.86 | 0.55 |
| hsa-miR-146b-5p | 18.19 | 18.25 | 17.55 | 17.79 | 17.95 | 0.34 |
| hsa-miR-411-5p | 17.54 | 18.80 | 18.11 | 17.73 | 18.04 | 0.56 |
| hsa-miR-30d-5p | 17.95 | 18.35 | 18.30 | 17.58 | 18.05 | 0.35 |
| hsa-miR-10b-5p | 17.60 | 18.62 | 18.51 | 17.52 | 18.06 | 0.58 |
| hsa-miR-495-3p | 17.80 | 17.79 | 18.06 | 18.60 | 18.06 | 0.38 |
| hsa-miR-424-5p | 17.97 | 18.75 | 17.52 | 18.04 | 18.07 | 0.51 |
| hsa-miR-654-5p | 18.41 | 17.97 | 18.03 | 18.11 | 18.13 | 0.20 |
| hsa-miR-30e-3p | 17.94 | 17.92 | 18.89 | 17.86 | 18.15 | 0.49 |
| hsa-miR-34c-5p | 17.74 | 18.07 | 18.87 | 17.99 | 18.17 | 0.49 |
| hsa-let-7g-5p | 18.19 | 18.56 | 17.87 | 18.13 | 18.19 | 0.29 |
| hsa-miR-30a-3p | 17.98 | 18.19 | 18.30 | 18.29 | 18.19 | 0.15 |
| hsa-miR-199b-5p | 18.10 | 18.68 | 18.24 | 18.07 | 18.27 | 0.28 |
| hsa-miR-34a-3p | 17.90 | 18.63 | 18.95 | 17.68 | 18.29 | 0.60 |
| hsa-miR-186-5p | 18.16 | 18.38 | 18.31 | 18.39 | 18.31 | 0.11 |
| hsa-miR-204-5p | 17.03 | 19.31 | 19.04 | 18.08 | 18.37 | 1.04 |
| hsa-miR-744-5p | 18.76 | 18.53 | 18.38 | 18.07 | 18.43 | 0.29 |
| mmu-miR-374-5p | 18.19 | 19.17 | 18.35 | 18.11 | 18.45 | 0.49 |
| hsa-miR-19a-3p | 18.37 | 17.97 | 18.54 | 18.99 | 18.46 | 0.42 |
| hsa-miR-212-3p | 18.56 | 18.82 | 18.47 | 18.36 | 18.55 | 0.20 |
| hsa-miR-103-3p | 18.77 | 18.98 | 18.46 | 18.09 | 18.57 | 0.39 |
| hsa-miR-125a-5p | 18.54 | 18.23 | 19.07 | 18.86 | 18.68 | 0.37 |
| hsa-miR-199a-5p | 18.70 | 19.46 | 18.45 | 18.27 | 18.72 | 0.52 |
| hsa-miR-10b-3p | 18.77 | 18.74 | 18.81 | 18.60 | 18.73 | 0.09 |
| hsa-miR-423-5p | 19.14 | 18.47 | 18.55 | 18.91 | 18.77 | 0.31 |
| hsa-miR-29a-5p | 18.85 | 18.99 | 18.89 | 18.50 | 18.81 | 0.21 |
| hsa-miR-410-3p | 18.44 | 19.17 | 18.63 | 19.03 | 18.82 | 0.34 |
| hsa-miR-7-1-3p | 18.92 | 18.70 | 18.95 | 19.06 | 18.91 | 0.15 |
| hsa-miR-532-3p | 18.80 | 19.07 | 19.28 | 18.58 | 18.94 | 0.31 |
| hsa-miR-1291 | 19.04 | 19.38 | 18.97 | 18.60 | 19.00 | 0.32 |
| hsa-miR-590-5p | 18.56 | 18.81 | 19.23 | 19.54 | 19.04 | 0.43 |
| hsa-miR-301a-3p | 18.76 | 18.75 | 18.83 | 19.81 | 19.04 | 0.52 |
| hsa-miR-134-5p | 18.86 | 18.38 | 19.60 | 19.40 | 19.06 | 0.55 |
| hsa-miR-494-3p | 18.94 | 18.81 | 18.77 | 19.73 | 19.06 | 0.45 |
| hsa-miR-137 | 19.74 | 19.47 | 18.57 | 18.91 | 19.17 | 0.53 |
| hsa-miR-503-5p | 19.07 | 19.85 | 19.27 | 19.82 | 19.50 | 0.39 |
| hsa-miR-379-5p | 19.41 | 19.85 | 19.59 | 19.64 | 19.62 | 0.18 |
| hsa-let-7d-5p | 19.83 | 20.25 | 19.75 | 19.08 | 19.73 | 0.48 |
| hsa-miR-34b-3p | 19.46 | 19.27 | 20.79 | 19.59 | 19.78 | 0.69 |
| hsa-miR-22-5p | 19.77 | 20.28 | 19.69 | 19.42 | 19.79 | 0.36 |
| hsa-miR-452-5p | 19.54 | 19.86 | 19.55 | 20.51 | 19.86 | 0.45 |
| hsa-miR-192-5p | 19.73 | 19.89 | 19.91 | 20.10 | 19.91 | 0.15 |
| hsa-miR-324-3p | 19.94 | 19.71 | 19.88 | 20.35 | 19.97 | 0.27 |
| hsa-miR-345-5p | 19.96 | 19.36 | 19.96 | 20.99 | 20.07 | 0.68 |
| hsa-miR-424-3p | 20.64 | 20.14 | 19.83 | 20.08 | 20.17 | 0.34 |
| hsa-miR-95-3p | 18.63 | 18.62 | 21.40 | 22.06 | 20.18 | 1.81 |
| hsa-miR-455-3p | 20.78 | 20.88 | 19.29 | 20.06 | 20.25 | 0.74 |
| hsa-miR-708-5p | 20.60 | 20.14 | 21.59 | 18.93 | 20.31 | 1.10 |
| hsa-miR-491-5p | 20.61 | 20.51 | 20.85 | 20.03 | 20.50 | 0.35 |
| hsa-miR-128a-3p | 20.62 | 20.30 | 20.49 | 21.04 | 20.61 | 0.31 |
| hsa-miR-222-5p | 20.66 | 20.40 | 21.02 | 20.86 | 20.73 | 0.27 |
| hsa-miR-185-5p | 20.69 | 20.50 | 20.71 | 21.22 | 20.78 | 0.31 |
| hsa-miR-330-3p | 20.82 | 20.85 | 20.54 | 20.93 | 20.79 | 0.17 |
| hsa-miR-664-3p | 20.94 | 20.97 | 20.82 | 20.50 | 20.81 | 0.22 |
| hsa-miR-323-3p | 20.64 | 20.01 | 21.00 | 21.61 | 20.81 | 0.67 |
| hsa-miR-15a-5p | 20.87 | 21.14 | 21.14 | 20.15 | 20.83 | 0.47 |
| hsa-miR-146a-5p | 21.97 | 19.85 | 20.16 | 21.34 | 20.83 | 0.99 |
| hsa-miR-148b-3p | 20.84 | 20.87 | 20.94 | 21.06 | 20.93 | 0.10 |
| hsa-miR-485-3p | 20.80 | 20.76 | 21.25 | 21.19 | 21.00 | 0.26 |
| hsa-miR-155-5p | 21.45 | 20.81 | 20.21 | 21.60 | 21.02 | 0.64 |
| hsa-miR-455-5p | 21.63 | 20.90 | 20.41 | 21.22 | 21.04 | 0.51 |
| hsa-miR-766-3p | 21.69 | 20.39 | 20.81 | 21.71 | 21.15 | 0.66 |
| hsa-miR-539-5p | 20.97 | 20.87 | 21.26 | 21.54 | 21.16 | 0.30 |
| hsa-miR-154-5p | 21.07 | 21.16 | 21.00 | 21.41 | 21.16 | 0.18 |
| hsa-miR-487b-3p | 20.84 | 20.88 | 21.22 | 21.95 | 21.22 | 0.51 |
| hsa-miR-320b | 21.34 | 21.34 | 21.37 | 21.02 | 21.27 | 0.16 |
| hsa-miR-433-3p | 21.55 | 20.91 | 21.48 | 21.51 | 21.36 | 0.30 |
| hsa-miR-193b-5p | 22.00 | 20.82 | 21.54 | 21.20 | 21.39 | 0.50 |
| hsa-miR-20b-5p | 22.25 | 20.03 | 21.13 | 22.20 | 21.40 | 1.05 |
| hsa-miR-203a-3p | 23.03 | 20.40 | 20.70 | 21.48 | 21.40 | 1.17 |
| hsa-miR-339-3p | 21.31 | 21.44 | 21.48 | 21.48 | 21.43 | 0.08 |
| hsa-miR-18a-5p | 21.69 | 20.55 | 21.38 | 22.12 | 21.43 | 0.66 |
| hsa-miR-337-5p | 21.12 | 21.38 | 21.52 | 21.87 | 21.47 | 0.31 |
| hsa-miR-638 | 21.72 | 21.43 | 21.65 | 21.15 | 21.49 | 0.26 |
| hsa-miR-543 | 21.73 | 21.30 | 21.57 | 21.60 | 21.55 | 0.18 |
| hsa-miR-24-2-5p | 21.43 | 21.83 | 21.77 | 21.19 | 21.55 | 0.30 |
| hsa-miR-184 | 21.06 | 22.64 | 21.58 | 21.06 | 21.58 | 0.75 |
| hsa-miR-505-3p | 22.03 | 21.40 | 21.11 | 21.83 | 21.59 | 0.41 |
| hsa-miR-942-5p | 21.72 | 21.33 | 21.97 | 21.96 | 21.74 | 0.30 |
| hsa-miR-502-3p | 21.83 | 21.68 | 22.30 | 22.06 | 21.97 | 0.27 |
| hsa-miR-140-3p | 21.65 | 22.38 | 22.05 | 21.97 | 22.01 | 0.30 |
| hsa-miR-381-3p | 21.79 | 22.25 | 21.99 | 22.10 | 22.03 | 0.19 |
| hsa-miR-125b-1-3p | 22.57 | 21.89 | 22.46 | 21.28 | 22.05 | 0.60 |
| hsa-miR-542-3p | 21.58 | 22.85 | 22.03 | 21.80 | 22.07 | 0.55 |
| hsa-miR-500a-5p | 21.95 | 22.32 | 22.26 | 21.75 | 22.07 | 0.27 |
| hsa-miR-190a-5p | 22.08 | 23.54 | 22.17 | 20.99 | 22.19 | 1.04 |
| hsa-miR-139-5p | 21.41 | 22.38 | 22.23 | 22.81 | 22.21 | 0.59 |
| hsa-miR-432-5p | 22.45 | 21.84 | 22.21 | 22.71 | 22.30 | 0.37 |
| hsa-miR-671-3p | 22.14 | 21.64 | 23.17 | 22.37 | 22.33 | 0.64 |
| hsa-miR-101-3p | 21.79 | 23.71 | 22.06 | 21.96 | 22.38 | 0.90 |
| hsa-miR-181c-5p | 22.77 | 23.24 | 22.19 | 21.52 | 22.43 | 0.74 |
| hsa-miR-1290 | 22.44 | 21.93 | 22.53 | 22.85 | 22.44 | 0.38 |
| hsa-miR-129-3p | 23.75 | 21.74 | 21.21 | 23.23 | 22.48 | 1.20 |
| hsa-miR-652-3p | 22.43 | 22.73 | 22.17 | 22.65 | 22.50 | 0.25 |
| hsa-miR-597-5p | 22.60 | 22.52 | 21.91 | 22.97 | 22.50 | 0.44 |
| hsa-miR-889-3p | 21.82 | 22.41 | 22.51 | 23.37 | 22.53 | 0.64 |
| hsa-miR-214-5p | 22.61 | 22.47 | 22.40 | 23.14 | 22.65 | 0.33 |
| hsa-miR-576-3p | 22.66 | 22.23 | 22.75 | 23.27 | 22.73 | 0.42 |
| hsa-miR-493-3p | 22.15 | 22.72 | 22.88 | 23.67 | 22.86 | 0.63 |
| hsa-miR-409-5p | 22.70 | 22.85 | 22.81 | 23.10 | 22.87 | 0.17 |
| hsa-miR-625-3p | 22.80 | 22.39 | 22.81 | 23.58 | 22.89 | 0.50 |
| hsa-miR-1271-5p | 22.70 | 22.34 | 22.86 | 23.68 | 22.90 | 0.57 |
| hsa-miR-642a-5p | 22.77 | 22.28 | 23.06 | 23.52 | 22.91 | 0.52 |
| hsa-miR-136-3p | 22.60 | 23.03 | 22.94 | 23.07 | 22.91 | 0.21 |
| hsa-miR-502-5p | 23.31 | 23.05 | 22.58 | 22.77 | 22.93 | 0.32 |
| hsa-miR-329-3p | 22.86 | 22.63 | 22.87 | 23.37 | 22.93 | 0.31 |
| hsa-miR-769-5p | 23.12 | 23.29 | 23.06 | 22.38 | 22.96 | 0.40 |
| hsa-miR-99b-3p | 23.04 | 23.17 | 23.42 | 22.58 | 23.05 | 0.35 |
| hsa-miR-758-3p | 22.97 | 22.79 | 22.87 | 23.63 | 23.07 | 0.38 |
| hsa-miR-301b-3p | 22.48 | 23.38 | 23.47 | 23.04 | 23.09 | 0.45 |
| hsa-miR-454-3p | 23.24 | 23.33 | 23.69 | 22.12 | 23.10 | 0.68 |
| hsa-miR-497-5p | 22.79 | 23.35 | 22.96 | 23.36 | 23.11 | 0.28 |
| hsa-miR-369-3p | 23.04 | 23.55 | 23.00 | 22.99 | 23.15 | 0.27 |
| hsa-miR-362-5p | 23.04 | 23.87 | 23.62 | 22.12 | 23.16 | 0.78 |
| hsa-miR-196b-5p | 23.06 | 23.85 | 23.21 | 23.02 | 23.28 | 0.38 |
| hsa-miR-645 | 23.61 | 23.48 | 23.85 | 22.23 | 23.29 | 0.72 |
| hsa-miR-93-3p | 23.52 | 22.58 | 23.14 | 23.97 | 23.30 | 0.59 |
| hsa-miR-194-5p | 23.18 | 23.81 | 23.19 | 23.13 | 23.33 | 0.32 |
| hsa-miR-548b-5p | 23.87 | 22.30 | 23.54 | 23.81 | 23.38 | 0.74 |
| hsa-miR-376b-3p | 23.25 | 23.62 | 23.11 | 23.67 | 23.41 | 0.27 |
| hsa-miR-299-5p | 23.34 | 23.63 | 22.89 | 23.87 | 23.43 | 0.42 |
| hsa-miR-150-5p | 23.52 | 22.92 | 24.18 | 23.13 | 23.44 | 0.55 |
| hsa-miR-126-3p | 24.04 | 24.06 | 22.98 | 22.68 | 23.44 | 0.72 |
| hsa-miR-1180-3p | 23.41 | 23.28 | 23.56 | 23.52 | 23.44 | 0.13 |
| hsa-miR-151a-5p | 23.35 | 24.01 | 23.79 | 22.69 | 23.46 | 0.58 |
| hsa-miR-34b-5p | 23.02 | 23.31 | 24.34 | 23.27 | 23.49 | 0.58 |
| hsa-miR-572 | 23.55 | 23.13 | 24.38 | 23.28 | 23.58 | 0.56 |
| hsa-miR-629-3p | 23.55 | 23.21 | 23.56 | 24.03 | 23.59 | 0.33 |
| hsa-miR-198 | 23.35 | 24.37 | 23.53 | 23.13 | 23.59 | 0.54 |
| hsa-miR-542-5p | 23.68 | 23.57 | 23.43 | 23.85 | 23.63 | 0.18 |
| hsa-miR-362-3p | 23.42 | 23.86 | 23.77 | 23.49 | 23.64 | 0.21 |
| hsa-miR-579-3p | 23.81 | 23.09 | 23.85 | 24.45 | 23.80 | 0.56 |
| hsa-miR-296-3p | 23.75 | 23.67 | 23.56 | 24.34 | 23.83 | 0.35 |
| hsa-miR-27a-5p | 24.23 | 23.56 | 24.13 | 23.66 | 23.90 | 0.34 |
| hsa-miR-1226-5p | 24.69 | 23.34 | 24.08 | 23.75 | 23.96 | 0.57 |
| hsa-miR-616-3p | 24.40 | 23.85 | 23.51 | 24.66 | 24.11 | 0.52 |
| hsa-miR-340-5p | 23.16 | 24.53 | 24.00 | 25.13 | 24.20 | 0.84 |
| hsa-miR-548d-5p | 24.55 | 23.59 | 24.32 | 24.83 | 24.32 | 0.53 |
| hsa-miR-628-5p | 23.90 | 24.79 | 24.26 | 24.43 | 24.34 | 0.37 |
| hsa-miR-605-5p | 24.86 | 23.38 | 24.02 | 25.23 | 24.37 | 0.84 |
| hsa-miR-181a-2-3p | 24.76 | 25.31 | 24.59 | 23.25 | 24.48 | 0.87 |
| hsa-miR-99a-3p | 24.52 | 24.40 | 24.76 | 24.49 | 24.54 | 0.16 |
| hsa-miR-27b-5p | 24.64 | 24.85 | 24.90 | 23.79 | 24.54 | 0.52 |
| hsa-miR-598-3p | 25.48 | 23.94 | 24.61 | 24.19 | 24.56 | 0.67 |
| hsa-miR-146b-3p | 24.91 | 24.61 | 23.89 | 25.31 | 24.68 | 0.60 |
| hsa-miR-548c-5p | 25.44 | 23.40 | 24.13 | 25.94 | 24.73 | 1.17 |
| hsa-miR-450a-5p | 24.53 | 25.18 | 24.25 | 25.09 | 24.76 | 0.45 |
| hsa-miR-380-5p | 24.97 | 24.13 | 24.60 | 25.40 | 24.78 | 0.54 |
| hsa-miR-1255b-5p | 25.48 | 23.32 | 24.21 | 26.30 | 24.83 | 1.32 |
| hsa-miR-654-3p | 25.31 | 24.69 | 24.47 | 25.04 | 24.88 | 0.37 |
| hsa-miR-337-3p | 24.69 | 24.98 | 24.67 | 25.38 | 24.93 | 0.33 |
| hsa-miR-425-3p | 25.27 | 25.26 | 25.41 | 24.22 | 25.04 | 0.55 |
| hsa-miR-145-3p | 24.71 | 26.05 | 24.43 | 25.10 | 25.07 | 0.71 |
| hsa-miR-451 | 24.62 | 25.84 | 25.51 | 25.03 | 25.25 | 0.54 |
| hsa-miR-505-5p | 25.63 | 25.54 | 24.87 | 25.04 | 25.27 | 0.37 |
| hsa-miR-616-5p | 25.67 | 25.06 | 25.28 | 25.72 | 25.43 | 0.32 |
| hsa-miR-23a-5p | 26.21 | 25.09 | 25.45 | 25.27 | 25.50 | 0.49 |
| hsa-miR-154-3p | 24.97 | 26.60 | 25.88 | 25.33 | 25.70 | 0.71 |
| hsa-miR-744-3p | 25.58 | 25.22 | 25.75 | 26.41 | 25.74 | 0.50 |
| hsa-miR-206 | 26.36 | 26.30 | 27.14 | 23.33 | 25.78 | 1.68 |
| hsa-miR-126-5p | 25.78 | 26.69 | 25.62 | 25.14 | 25.81 | 0.65 |
| hsa-miR-19b-1-5p | 26.03 | 25.70 | 26.06 | 25.70 | 25.87 | 0.20 |
| hsa-miR-624-5p | 26.81 | 25.54 | 25.84 | 25.68 | 25.97 | 0.57 |
| hsa-miR-10a-3p | 26.70 | 26.37 | 25.80 | 25.56 | 26.11 | 0.52 |
| hsa-miR-30d-3p | 26.87 | 26.42 | 25.98 | 26.30 | 26.39 | 0.37 |
| hsa-miR-148b-5p | 26.28 | 26.91 | 26.70 | 25.79 | 26.42 | 0.50 |
| hsa-miR-622 | 27.69 | 26.41 | 26.69 | 25.45 | 26.56 | 0.92 |
| hsa-miR-377-5p | 26.34 | 27.35 | 26.10 | 26.59 | 26.60 | 0.54 |
| hsa-miR-378-5p | 26.39 | 26.03 | 27.62 | 26.49 | 26.64 | 0.69 |
| hsa-miR-221-5p | 26.43 | 27.34 | 26.88 | 26.26 | 26.73 | 0.48 |

Yellow background for miRNAs in the first quartile of abundance
