# Supplementary material for: Secreted Factors and EV-miRNAs Orchestrate the Healing Capacity of Adipose Mesenchymal Stem Cells for the Treatment of Knee Osteoarthritis
Source: Int J Mol Sci. 2020 Feb 26;21(5):1582. doi: 10.3390/ijms21051582 (PMC7084308; doi:10.3390/ijms21051582)
Supplement: Supplementary file 1 [file ijms-21-01582-s001.zip › Supplementary Table 2_IJMS.docx]

Supplementary Table 2. Target mRNAs for 65 EV-miRNAs in the first quartile of expression

| AARSD1 | ARL2 | CADM1 | CDK4 | CSNK1D | EIF4E | FSCN1 | HK2 | IPO4 | LYPLA2 |
| --- | --- | --- | --- | --- | --- | --- | --- | --- | --- |
| ABCB1 | ARPC3 | CALCOCO2 | CDK5RAP1 | CTNNBIP1 | EIF4EBP2 | FUBP1 | HMGA1 | IRS1 | MAD2L1 |
| ABCF2 | ASXL2 | CALU | CDK6 | CTNND1 | EIF4G2 | FURIN | HMGA2 | ISCU | MAFB |
| ABCG2 | ATAD3B | CAMTA1 | CDKAL1 | CXCL8 | ELAVL1 | FXR2 | HMGN3 | ITCH | MAN1A1 |
| ABHD10 | ATF4 | CAPG | CDKN1A | CYP1A1 | ELM0D2 | GAK | HMOX1 | ITGA2 | MAP2 |
| ABRACL | ATF6 | CAPN8 | CDKN1B | CYP1B1 | ENPP6 | GALNT1 | HNRNPM | ITGA5 | MAP2K1 |
| ABTB1 | ATG2B | CAPRIN1 | CDKN1C | DAD1 | ENTPD4 | GALNT13 | HOTAIR | ITGB3 | MAP2K4 |
| ACBD3 | ATG9A | CARD8 | CDKN2A | DCP2 | EPHA2 | GALNT7 | HOXA1 | JAG1 | MAP2K7 |
| ACP1 | ATP2A2 | CARHSP1 | CEBPG | DDIT4 | ERBB2 | GAS7 | HOXÅ1 | JAK1 | MAP3K12 |
| ACP2 | ATP6AP1L | CASP3 | CEMIP2 | DDR1 | ERBB3 | GCA | HOXA5 | JARID2 | MAP4K4 |
| ACSS1 | ATP6V1F | CASP6 | CENPJ | DDX19B | ERBB4 | GEM1 | HOXD10 | JUN | MAPK14 |
| ACTA2 | ATP6VOA1 | CASP7 | CEP63 | DFFA | ESR1 | GEMIN7 | HPF1 | JUNB | MAPK3 |
| ACTR1A | ATRX | CASP8AP2 | CEP72 | DHER | ETS1 | GFPT1 | HPGD | KCNJ16 | MAPK7 |
| ACVR1 | AURK8 | CASR | CES1 | DHX57 | EZH2 | GLCCI1 | HSDL2 | KCNN4 | MAPRE1 |
| ACVR1B | AURKB | CAV2 | CFL2 | DICER1 | F11R | GMFB | HSP90B1 | KCTD3 | MAPRE2 |
| ACVR2A | AXIN2 | CBCA5 | CHDI | DICERI | F2 | GNAI2 | HSPA1A | KDELC2 | MARCKS |
| ADAMTS1 | B3GALT4 | CBFB | CHEK1 | DIO3 | FADD | GNL3L | HSPB6 | KIF23 | MARS2 |
| ADGRG1 | BACE1 | CBKN1C | CHMP2A | DIPK1A | FADS2 | GOLGA5 | HTR1A | KIT | MAT2A |
| ADORA2B | BAK1 | CBLN2 | CHORDC1 | DIRAS3 | FAM122C | GOLPH3L | HYAL3 | KITLG | MAX |
| ADPGK | BAX | CBX7 | CIAO2A | DLL1 | FAM3C | GPAM | HYOU1 | KLF13 | MAZ |
| ADSS | BBC3 | CCDC25 | CLDN12 | DMTF1 | FANCD2 | GPD1 | ICAM1 | KLF4 | MBNL1 |
| AG04 | BCL2 | CCKBR | CLINTI | DNAJB4 | FAS | GPD2 | ID1 | KLF5 | MCL1 |
| AHNAK | BCL2L1 | CCL6 | CNOT8 | DNMT1 | FASLG | GPR160 | ID2 | KLK1 | MDFI |
| AJUBA | BCL2L11 | CCL9 | CNOT9 | DNMT3A | FBN1 | GPR37 | ID3 | KLK10 | MDM2 |
| AKAP8 | BCL2L2 | CCN2 | CNTN4 | DNMT3B | FBXO28 | GRB10 | IDHI | KPNA3 | MECP2 |
| ALCAM | BCL6 | CCN5 | COIL | DOCK5 | FBXW7 | GRB2 | IFI16 | KRAS | MED28 |
| ALD1HA2 | BCL7A | CCNA2 | COK6 | DOCK7 | FEN1 | GRPEL2 | IFIT5 | KRT19 | MEF2C |
| ALOX5 | BDNF | CCND1 | COL15A1 | DRD3 | FGF16 | GSK38 | IFRD1 | KRT7 | MEF2D |
| ANAPC1 | BECN1 | CCND3 | COL1A1 | DSP | FGF2 | GSS | IFRDI | KRT85 | MEOX2 |
| ANAPC16 | BMF | CCNDI | COL1A2 | DTD1 | FGF7 | GSTM4 | IGF1 | LAMB | MET |
| ANLN | BMI1 | CCNE1 | COL3A1 | DUS1L | FGFR1 | GTF2H1 | IGF1R | LAMC1 | MGAT4A |
| ANPEP | BMP2K | CCNE2 | COL4A1 | DUSP12 | FGFR3 | GTPBP3 | IGF2BP1 | LAMP2 | MICA |
| AP15 | BMP7 | CCNF | COL4A2 | DUSP2 | FGFRL1 | GYS1 | IGF2BP2 | LAMTOR3 | MIR9 |
| AP2A1 | BMPR18 | CCR5 | COL5A2 | DUSP23 | FL1 | H3F3A | IGF2BP3 | LAMTOR5 | MLF1 |
| AP2M1 | BMPR2 | CD276 | COL5A3 | E2F1 | FLT3 | HACE1 | IGF2R | LATS2 | MLLT1 |
| APAF1 | BNIP2 | CD44 | COMMD9 | E2F2 | FN1 | HARS | IGFBP3 | LDAH | MLLT11 |
| APLN | BNIP3L | CD47 | CPNE8 | E2F3 | FNDC3A | HBP1 | IKZF1 | LIN28A | MMP1 |
| APP | BRCA1 | CDC14A | CREB1 | E2F5 | FNDC3B | HDAC1 | IKZF4 | LIPA | MMP13 |
| AR | BSG | CDC14B | CREBL2 | E2F6 | FOS | HDAC4 | IL10 | LMNB2 | MMP3 |
| ARAF | BTG2 | CDC25A | CRHBP | ECHDC1 | FOXO1 | HDHD2 | IL1RN | LOXL2 | MMP9 |
| ARHGAP32 | C11orf58 | CDC42 | CRIM1 | EFNA3 | FOXO3 | HERC6 | IL2RG | LRRC8C | MNT |
| ARHGDIA | C17orf80 | CDCP1 | CRP | EGER | FOXP1 | HGS | IL6 | LRRFIP1 | MPDU1 |
| ARID38 | C2orf74 | CDH5 | CSDE1 | EGFR | FOXP3 | HIF1A | IL6R | LTN1 | MRM1 |
| ARID3A | CA12 | CDIPT | CSF1 | EGR2 | FRAT2 | HIPK3 | ING4 | LUZP1 | MRPL20 |
| ARID4B | CACNA2D1 | CDK1 | CSHL1 | EIF3J | FRG1 | HIST1H4A | INSIG1 | LYPLA1 | MRPS24 |

| MRPS33 | ONECUT2 | POLD2 | RAD52 | SCYL1 | SMOX | TGFBR2 | TSPAN3 | ZBTB7A |
| --- | --- | --- | --- | --- | --- | --- | --- | --- |
| MSH2 | OSBPL2 | POLR2C | RAF1 | SDHD | SNAP23 | TGM2 | TSPANB | ZEB2 |
| MTAP | OSBPL8 | POM1 | RAP1A | SEC23A | SOCS5 | TH8S1 | TTC9C | ZFP36L1 |
| MTOR | OSBPL9 | PON2 | RAP1B | SEC24A | SOD2 | THBS1 | TUBB2A | ZFPM2 |
| MTPN | OSGEPL1 | POU4F2 | RARS | SEC62 | SOD3 | THEM4 | TUSC2 | ZNF385A |
| MTRR | OTULINL | PPARA | RAS | SEPT3 | SOX4 | THRB | TXN2 | ZNF559 |
| MUC1 | P4HA2 | PPARG | RASA1 | SERPINB5 | SOX5 | TIA1 | TYMS | ZNF622 |
| MYB | PADI1 | PPIC | RB1 | SERPINE2 | SP1 | TIMP3 | UAP1 | ZYX |
| MYBL2 | PAFAH1B2 | PPIF | RBL2 | SESN1 | SPARC | TJP1 | UBE21 |  |
| MYC | PAK5 | PPM1D | RBM19 | SGK3 | SPCS3 | TLR3 | UBE2S |  |
| MYCN | PANX1 | PPP1R7 | RBM8A | SGPL1 | SPI1 | TLR4 | UBE4A |  |
| MYD88 | PARP8 | PPP2R2A | RBMS1 | SHOC2 | Spry1 | TLR7 | UCP2 |  |
| MYLIP | PCDHB10 | PPP2R5C | RDH10 | SHROOM2 | SPRY2 | TMCO1 | UGDH |  |
| MYO10 | PCGF1 | PPP3CA | RECK | SIGMAR1 | SPRYD4 | TMED10 | UGP2 |  |
| NAA15 | PCTP | PPP3R1 | RERE | SIRT1 | SPTB | TMED2 | UGT2B15 |  |
| NAPG | PDCD4 | PPT2 | RFFL | SKAP2 | SPTLC1 | TMED3 | UGT2B17 |  |
| NASP | PDCD6IP | PRDM1 | RFT1 | SLC12A1 | SQSTM1 | TMED7 | UGT2B28 |  |
| NAV3 | PDGFB | PRDX6 | RHEBL1 | SLC12A2 | SRF | TMEM109 | UGT8 |  |
| NCEH1 | PDPK1 | PRIM1 | RHOB | SLC12A4 | SRM | TMEM189 | UHRF1 |  |
| NCL | PELI1 | PRIMPOL | RHOC | SLC16A10 | SRPRA | TMEM251 | UNG |  |
| NCOA3 | PERP | PRPF40A | RHOG | SLC16A3 | SRPRB | TMEM41B | USF2 |  |
| NDUFA4 | PEX11B | PRRC2A | RHOT1 | SLC1A4 | SRSF10 | TMEM43 | USP46 |  |
| NEDD4 | PEX7 | PRRG4 | RICTOR | SLC25A1 | ST14 | TMEM59 | UTP15 |  |
| NEUROD1 | PGC | PSAT1 | RIDA | SLC25A13 | ST18 | TMEM87A | UVRAG |  |
| NF2 | PGM1 | PTEN | RNASEL | SLC25A22 | STAT3 | TNAIFP2 | VASN |  |
| NFIA | PGRMC1 | PTGFRN | ROBO4 | SLC25A24 | STK40 | TNF | VAV2 |  |
| NFIB | PHB | PTGS2 | ROCK2 | SLC25A32 | STRN | TNFAIP3 | VCAN |  |
| NIPAL2 | PHF6 | PTK2 | RP2 | SLC35A1 | STX1A | TNFRSF10B | VEGFA |  |
| NOTCH1 | PHKB | PTPA | RPP38 | SLC35B3 | STX7 | TNFS9 | VIM |  |
| NOTCH2 | PHLD82 | PTPN1 | RPS6KA5 | SLC38A1 | SUZ12 | TNRC6A | VOPP1 |  |
| NPR3 | PIGR | PTPRK | RPS6KB2 | SLC38Å1 | SWAP70 | TOMM34 | VPS39 |  |
| NPTX1 | PIK3C2A | PTPRM | RPTOR | SLC38A5 | SYPL1 | TOR2A | VPS45 |  |
| NR1I2 | PIK3R1 | PTRH1 | RRP8 | SLC45A3 | SYT4 | TP53 | VSIR |  |
| NR4A2 | PIK3R3 | PURA | RTCA | SLC4A10 | TAC1 | TP53I11 | VSNL1 |  |
| NRAS | PISD | PWWP2A | RTKN | SLC4A7 | TAFA1 | TP63 | VTI1B |  |
| NT5C3A | PKD2 | PXDN | RTL1 | SLC7A1 | TAGLN | TPI1 | WEE1 |  |
| NT5DC1 | PKMYT1 | PXN | RTN4 | SLC7A11 | TBK1 | TPM1 | WIPF1 |  |
| NT5E | PLAG1 | RAB21 | RUNX1 | SLC9A3R2 | TCF21 | TPM2 | WNT1 |  |
| NUCB1 | PLAU | RAB27B | RUNX2 | SMAD1 | TCL1A | TPM3 | WNT3A |  |
| NUFIP2 | PLCG1 | RAB30 | RXRA | SMAD3 | TDG | TPPP3 | WNT5A |  |
| NUMBL | PLK1 | RAB9B | S1PR1 | SMAD4 | TENM2 | TRIM71 | WT1 |  |
| NXN | PMAIP1 | RABGAP1L | SATB2 | SMAD5 | TERT | TRIM9 | XBP1 |  |
| ODCl | PMS1 | RABL6 | scD | SMARCA5 | TET1 | TRMT1 | YIF1B |  |
| OGT | PNN | RAD23B | SCN3A | SMC1A | TGFB3 | TRMT13 | YY1 |  |
| OMA1 | PNP | RAD51C | SCRIB | SMO | TGFBR1 | TRPS1 | ZBTB10 |  |
