# Supplementary material for: Secreted Factors and EV-miRNAs Orchestrate the Healing Capacity of Adipose Mesenchymal Stem Cells for the Treatment of Knee Osteoarthritis
Source: Int J Mol Sci. 2020 Feb 26;21(5):1582. doi: 10.3390/ijms21051582 (PMC7084308; doi:10.3390/ijms21051582)
Supplement: Supplementary file 1 [file ijms-21-01582-s001.zip › Supplementary Table 3_IJMS.docx]

Supplementary Table 3. Target mRNAs for 65 EV-miRNAs in the first quartile of expression that were filtered through highly expressed OA-cartilage genes

| ACBD3 | DOCK7 | KDELC2 | PPP2R5C | SMARCA5 | VPS39 |
| --- | --- | --- | --- | --- | --- |
| ACVR1 | DSP | KITLG | PPP3CA | SMC1A | WIPF1 |
| ADSS | EIF4EBP2 | KLF4 | PRDX6 | SOD2 | XBP1 |
| AHNAK | EIF4G2 | LAMC1 | PRPF40A | SOD3 | ZFP36L1 |
| ANAPC16 | ENTPD4 | LAMP2 | PRRC2A | SOX4 |  |
| AP2M1 | ETS1 | LOXL2 | PTEN | SOX5 |  |
| APP | FBN1 | LRRFIP1 | PTK2 | SP1 |  |
| ARID4B | FGF2 | LTN1 | PTPRM | SPARC |  |
| ATF4 | FGFR1 | MAN1A1 | PURA | SPCS3 |  |
| ATF6 | FGFR3 | MAP4K4 | PXDN | SQSTM1 |  |
| ATG2B | FGFRL1 | MAPRE2 | RABGAP1L | SRSF10 |  |
| ATP2A2 | FN1 | MAT2A | RAD23B | STAT3 |  |
| ATRX | FNDC3A | MBNL1 | RAF1 | STRN |  |
| BACE1 | FNDC3B | MCL1 | RARS | STX7 |  |
| BCL2 | FOS | MDM2 | RASA1 | TET1 |  |
| BCL6 | FOXO1 | MECP2 | RBL2 | TGFBR1 |  |
| BMPR2 | FOXO3 | MEF2C | RBMS1 | TGFBR2 |  |
| BNIP2 | FOXP1 | MEF2D | RERE | THBS1 |  |
| BNIP3L | FSCN1 | MLLT1 | RHOB | THRB |  |
| BSG | FUBP1 | MMP3 | RICTOR | TIA1 |  |
| C11orf58 | FURIN | MYO10 | ROCK2 | TIMP3 |  |
| CALCOCO2 | GALNT1 | NASP | RTKN | TMCO1 |  |
| CALU | GAS7 | NCL | RTN4 | TMED10 |  |
| CAPG | GFPT1 | NCOA3 | RUNX1 | TMED2 |  |
| CAPRIN1 | GNAI2 | NFIA | RXRA | TMED3 |  |
| CCND1 | GNL3L | NFIB | scD | TMED7 |  |
| CD44 | GOLGA5 | NOTCH2 | SEC23A | TMEM43 |  |
| CD47 | GPD2 | NT5E | SEC24A | TMEM59 |  |
| CDC42 | HIF1A | NUCB1 | SEC62 | TMEM87A |  |
| CDK6 | HIPK3 | NUFIP2 | SERPINE2 | TNRC6A |  |
| CEP63 | HNRNPM | OGT | SLC12A4 | TPI1 |  |
| CNOT8 | HSP90B1 | OSBPL8 | SLC16A3 | TPM2 |  |
| COL15A1 | HYOU1 | OSBPL9 | SLC1A4 | TPM3 |  |
| COL1A1 | ID1 | P4HA2 | SLC25A1 | TRPS1 |  |
| COL1A2 | ID2 | PAFAH1B2 | SLC25A13 | TSPAN3 |  |
| COL3A1 | ID3 | PDCD4 | SLC25A22 | UAP1 |  |
| COL5A2 | IGF2R | PDCD6IP | SLC25A24 | UBE4A |  |
| CRIM1 | INSIG1 | PGM1 | SLC25A32 | UGDH |  |
| CSDE1 | ITCH | PGRMC1 | SLC35A1 | UGP2 |  |
| CYP1B1 | ITGA5 | PIK3C2A | SLC35B3 | VASN |  |
| DAD1 | JAG1 | PIK3R1 | SLC38A1 | VCAN |  |
| DDIT4 | JAK1 | PKD2 | SMAD3 | VEGFA |  |
| DICER1 | JUNB | PNN | SMAD4 | VIM |  |
| DMTF1 | KCTD3 | PPIC | SMAD5 | VOPP1 |  |
