# Supplementary figures and images for: Secreted Factors and EV-miRNAs Orchestrate the Healing Capacity of Adipose Mesenchymal Stem Cells for the Treatment of Knee Osteoarthritis
Source: Int J Mol Sci. 2020 Feb 26;21(5):1582. doi: 10.3390/ijms21051582 (PMC7084308; doi:10.3390/ijms21051582)

## Slide 1
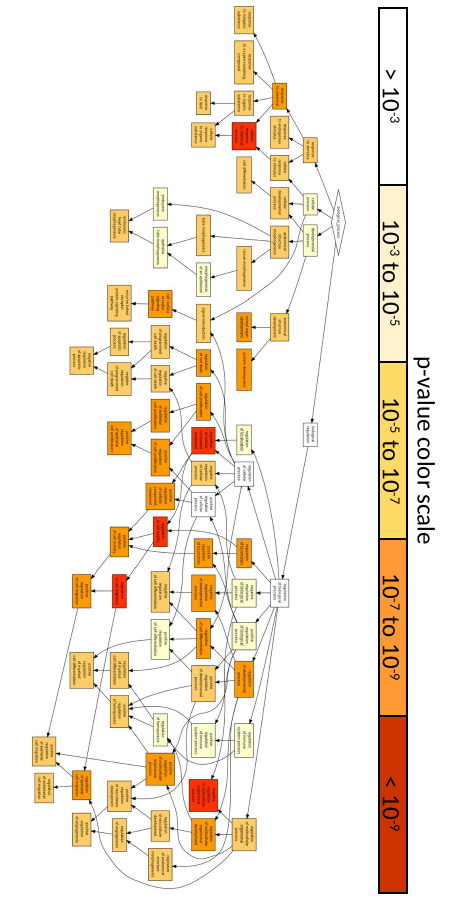

p-value color scale
> 10-3
10-3 to 10-5
10-5 to 10-7
10-7 to 10-9
< 10-9

Supplement: Supplementary file 1 [file ijms-21-01582-s001.zip › Supplementary Figure 1_ IJMS.pptx]

## Slide 1
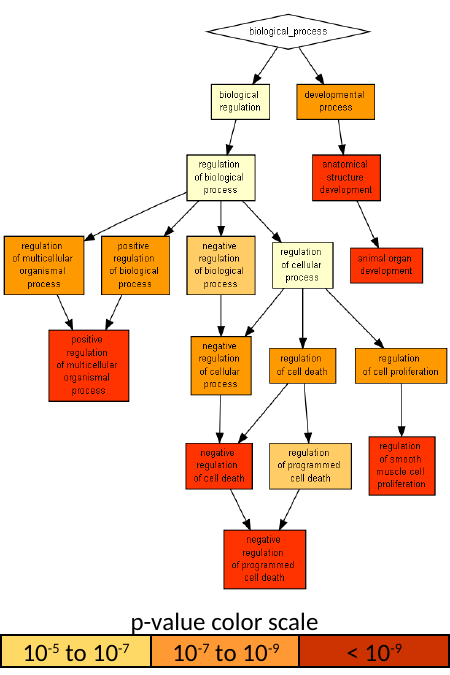

p-value color scale
10-5 to 10-7
10-7 to 10-9
< 10-9

Supplement: Supplementary file 1 [file ijms-21-01582-s001.zip › Supplementary Figure 2_ IJMS.pptx]
